# Responses of the Differentiated Intestinal Epithelial Cell Line Caco-2 to Infection With the *Giardia intestinalis* GS Isolate

**DOI:** 10.3389/fcimb.2018.00244

**Published:** 2018-07-16

**Authors:** Showgy Y. Ma'ayeh, Livia Knörr, Karin Sköld, Alexandra Garnham, Brendan R. E. Ansell, Aaron R. Jex, Staffan G. Svärd

**Affiliations:** ^1^Department of Cell and Molecular Biology, Uppsala University, Uppsala, Sweden; ^2^Population Health & Immunity Division, The Walter and Eliza Hall Institute of Medical Research, Parkville, VIC, Australia; ^3^Faculty of Veterinary Science, The University of Melbourne, Parkville, VIC, Australia

**Keywords:** host-parasite interaction, *Giardia*, transcriptome, cell cycle, apoptosis

## Abstract

*Giardia intestinalis* is a parasitic protist that causes diarrhea in humans, affecting mainly children of the developing world, elderly and immunocompromised individuals. Humans are infected by two major *Giardia* assemblages (i.e. genetic subtypes), A and B, with the latter being the most common. So far, there is little information on molecular or cellular changes during infections with assemblage B. Here, we used RNA sequencing to study transcriptional changes in Caco-2 intestinal epithelial cells (IECs) co-incubated with assemblage B (GS isolate) trophozoites for 1.5, 3, and 4.5 h. We aimed to identify early molecular events associated with the establishment of infection and followed cellular protein changes up to 10 h. IEC transcriptomes showed a dominance of immediate early response genes which was sustained across all time points. Transcription of inflammatory cytokines (e.g., *cxcl1-3, ccl2, 1l1a*, and *il1b*) peaked at 1.5 and 3 h of infection. Compared to co-incubation with assemblage A *Giardia*, we identified the induction of novel cytokines (*cxcl8, cxcl10, csf1, cx3cl1, il12a, il11*) and showed that inflammatory signaling is mediated by Erk1/2 phosphorylation (mitogen activated protein kinase, MAPK), nuclear factor kappa B (NFκB) and adaptor protein-1 (AP-1). We also showed that GS trophozoites attenuate P38 (MAPK) phosphorylation in IECs. Low amounts of IL-8, CXCL1 and CCL20 proteins were measured in the interaction medium, which was attributed to cytokine degradation by trophozoite secreted proteases. Based on the transcriptome, the decay of cytokines mRNA mediated by zinc finger protein 36 might be another mechanism controlling cytokine levels at later time points. IEC transcriptomes suggested homeostatic responses to counter oxidative stress, glucose starvation, and disturbances in amino acid and lipid metabolism. A large group of differentially transcribed genes were associated with cell cycle arrest and induction of apoptosis, which was validated at protein level. IEC transcriptomes also suggested changes in tight junction's integrity, microvilli structure and the extracellular mucin layer. This is the first study to illuminate transcriptional and protein regulatory events underlying IECs responses and pathogenesis during *Giardia* assemblage B infection. It highlights differences compared to assemblage A infections which might account for the differences observed in human infections with the two assemblages.

## Introduction

*Giardia intestinalis* is a parasitic protist that infects the small intestines of mammals, including humans (Thompson, 2000). In humans, infections are either asymptomatic or manifest as an acute diarrheal disease, which can develop to a chronic stage (Wolfe, 1978; Farthing, 1996). Giardiasis has a global distribution with 280 million symptomatic cases reported annually and is endemic in the developing world (Lane and Lloyd, 2002). Children living in poor socioeconomic conditions are the most affected with noticeable effects on nutrition, growth and cognitive function (Simsek et al., 2004; Prado et al., 2005; Nematian et al, 2008). Since giardiasis increases the global burden of parasitic diseases, it was included in the “Neglected Disease Initiative” by the World Health Organization in 2004, which aimed at launching a comprehensive approach for disease control and prevention (Savioli et al., 2006).

*Giardia* has two life-cycle stages; the cyst and the trophozoite. Infection starts with the ingestion of cysts in contaminated food and water. Cysts hatch in the small intestines releasing trophozoites, the etiologic agent of giardiasis (Thompson, 2004). Trophozoites possess a unique structure, the ventral disc, which allows parasite attachment to intestinal epithelial cells (IECs). This begins a process of interaction with host cells, leading to cellular damage, change in cell size and shape (Chávez et al., 1995; Teoh et al., 2000; Humen et al., 2011; Maia-Brigagão et al., 2012), cell cycle arrest (Stadelmann et al., 2012), induction of apoptosis (Chin et al., 2002; Panaro et al., 2007), villus atrophy (Buret et al., 1990; Panaro et al., 2007), digestive enzyme deficiencies (Humen et al., 2011) and electrolytes imbalance (Buret et al., 1990), which collectively lead to dysfunctional intestinal epithelial barrier (Cotton et al., 2011). These pathophysiological changes result in diarrhea and other associated symptoms (e.g., bloating, abdominal pain, nausea and vomiting). A large body of evidence relates these changes to trophozoite attachment (Humen et al., 2011) and parasite secreted proteins (SPs) such as cysteine proteases and metabolic enzymes (e.g., arginine deiminase, ornithine carbamoyl transferase and carbamate kinase) (Rodríguez-Fuentes et al., 2006; Ringqvist et al., 2008). Nevertheless, the information on disease mechanisms and parasite pathogenic factors are still incomplete.

Current molecular phylogenies have placed *G. intestinalis* isolates into eight distinct assemblages (i.e., genetic subtypes) designated from A to H (Yaoyu and Xiao, 2011; Cacciò et al., 2017). Humans are infected with parasites that belong only to assemblages A and B (Thompson, 2004), of which isolates WB and GS, respectively, are widely studied and their genomes have been sequenced and annotated (Morrison et al., 2007; Franzén et al., 2009; Jerlström-Hultqvist et al., 2010). Comparative genomics showed that these two isolates are different by 23% at nucleotide level and 22% at amino acid level (Franzén et al., 2009) and these differences have been proposed to account for variations in growth rate, infectivity and pathogenicity (Cacciò et al., 2017). For example, the GS isolate grows slower in axenic cultures (Karanis and Ey, 1998), but it is the only isolate capable of infecting adult mice without antibiotics pre-treatment (Byrd et al., 1994). The GS isolate established symptomatic infections in human volunteers whereas the assemblage A isolate Isr failed to establish an infection (Nash et al., 1987). In Mongolian gerbils, GS induces greater brush border enzyme deficiency, epithelial cell shortening, infiltration of higher numbers of inflammatory cells into the duodenum and jejunum, and consistent softening of feces (Bénéré et al., 2012). *In vitro*, GS isolate or its SPs produced stronger inflammatory responses in human IECs (HT-29), represented by the production of interleukins 8 and 1-beta (IL-8 and IL1-β) as well as tumor necrosis factor alpha (TNF-α) (Lee et al., 2012). Therefore, the research above indicates that the genetic variation between *Giardia* isolates, and more likely between assemblages, could account for the different cellular, immunological and pathological outcomes during infections.

Tissue culture models of giardiasis provide important information on disease mechanisms. Given the fact that IECs can be readily co-incubated with *Giardia* trophozoites, changes in gene transcription or protein expression can be monitored directly at cellular level. In a previous study using microarrays (Roxström-Lindquist et al., 2005), analysis of global changes in gene transcription in differentiated IECs (e.g., Caco-2) co-incubated with the WB isolate showed that, as early as 1.5 h of interaction, *Giardia* induces the transcription of chemokine ligand genes (*ccl20, ccl2, cxcl1-3*) whose products are required for attracting immune cells to the site of infection. IECs also up-regulated the transcription of stress and hypoxia genes [e.g., c-Fos and c-Jun, immediate-early response 3 (IER3), hypoxia-inducible factor 2 (HIG2)] and at a later stage of interaction (e.g., 18 h), the transcriptional changes showed a reduction in inflammatory gene transcription and cellular proliferation (Roxström-Lindquist et al., 2005). Thus, while IECs initiate immune signaling in response to *Giardia*, parasite persistence on the cell surface appears to cause stress (e.g., potentially via nutrient and arginine depletion, and the production of reactive oxygen species) and cell cycle arrest.

Studies of host-parasite interactions are important for understanding host defenses and the pathogenicity of the infecting isolate. Currently, there is no available information on IECs response to co-incubation with assemblage B *Giardia* isolates and the extent of difference to that in infections with assemblage A. Therefore, we co-incubated differentiated Caco-2 cells with GS isolate trophozoites *in vitro* and studied differential gene transcription via RNA sequencing. Transcriptional changes in both differentiated and non-differentiated (i.e., proliferating) Caco-2 cells were also compared to assess whether the differential status of the IECs could impact on cell response to parasitism. We focused on host cell responses during the early hours of interaction, as this period is important for the establishment of infection. The cellular events were followed at the protein level (up to 10 h) to verify important changes (e.g., in immune and signaling pathways, cell cycle arrest and the induction of apoptosis) in response to GS trophozoites.

## Materials and methods

### *Giardia* culture

*Giardia intestinalis* isolate GS, clone H7 (ATCC50581), was used in the experiments. Trophozoites were cultured in 10 or 50 ml Falcon tubes filled with TYDK medium prepared according to Keister (Keister, 1983) and supplemented with 10% heat inactivated bovine bile (Gibco, Thermo Fisher Scientific, MA, USA). Cultures were incubated at 37°C until confluence, upon which they were harvested for the experiments. All the components used in TYDK medium were purchased from Sigma-Aldrich (MO, USA) unless otherwise stated.

### Human intestinal epithelial cells

The human colon adenocarcinoma cell line (Caco-2, clone TC7) (Roxström-Lindquist et al., 2005) was used in the experiments at either differentiated or proliferating state (Passage no. 10–15). Caco-2 cells were cultured in 25 or 75 cm^2^ flasks as well as in chamber slides (Nunc® Lab-Tek® II, Corning, Sigma-Aldrich, MO, USA), all of which were supplied with complete Dulbecco's Modified Eagle Medium (DMEM), containing 10% fetal bovine serum (FBS), 2 mM GlutaMAX (Gibco, Thermo Fisher Scientific, MA, USA), 1x MEM non-essential amino acid solution (Sigma-Aldrich, MO, USA), 1x of penicillin-streptomycin 100x solution (10,000 units penicillin and 10 mg streptomycin/ml) (Sigma-Aldrich, MO, USA). For host-parasite interaction experiments, the FBS in DMEM was replaced with heat inactivated (HI)-FBS (Gibco, Thermo Fisher Scientific, MA, USA) to avoid adverse effects of active serum components on trophozoites' viability. All culture flasks including chamber slides were incubated in humidified 10% CO_2_ incubator at 37°C and used for the experiments either upon reaching confluence (i.e., proliferating) or at 21 days post-confluence (i.e., differentiated). Media was changed twice weekly during the differentiation process.

### Host-parasite interaction experiments

Differentiated Caco-2 cells were washed twice with warm PBS (37°C) and incubated with fresh DMEM supplemented with HI-FBS prior to the addition of trophozoites (10% CO_2_, 37°C) (10 ml for controls flask and 9 ml for test conditions). TYDK media in parasite cultures (10 or 50 ml tubes) were decanted and the tubes were washed once with warm PBS (37°C) to remove dead cells, then filled with ice cold PBS and incubated on ice for 10 min to detach trophozoites. Trophozoites were counted using a Neubauer Chamber slide and 1.5 × 10^7^ cells were pelleted by centrifugation (750 xg, 7 min, 4°C), resuspended in 1 ml of complete DMEM and added to culture flasks with 9 ml of media (T25 flasks, either at proliferating or differentiated state). Trophozoites were allowed to interact with IECs for 1.5, 3 and 4.5 h (10% CO_2_ at 37°C). Caco-2 cells incubated alone in DMEM served as a negative control. After incubation, the media were collected and the flasks were incubated with two rounds of 10 ml of ice-cold DMEM and placed on ice (10 and 3 min, respectively) to detach trophozoites. Following this step, cells were directly lysed in a lysis buffer provided in the PureLink® RNA Mini Kit (Ambion, Thermo Fisher Scientific, MA, USA) and stored at −80°C until RNA extraction. The collected spent culture media were centrifuged (1500 x*g*, 4°C, 7 min), filter sterilized (0.45 μm) and aliquoted into sterile Eppendorf tubes. Aliquots were stored at −80°C to be used for subsequent cytokine measurements.

### RNA extraction and RNA sequencing

Parasitized cells and control samples collected in lysis buffer (previous section) were processed with the PureLink® RNA Mini Kit according to the manufacturer's instruction. A DNase I treatment step was incorporated into the procedure to remove contaminating genomic DNA and was performed on the spin column during RNA extraction. The quality of extracted RNA was checked by measuring the 260/280 and 260/230 ratios using a NanoDrop 1000 Spectrophotometer (Thermo Fisher Scientific) and by running the samples (500 ng) on a 1.5% Tris-Borate-EDTA (TBE) agarose gel prepared with 20 mM of guanidium isothiocyanate (GITC). Samples were subjected to amplicon RNA sequencing using the Ion Proton Platform. This approach has great sensitivity to detect lowly-abundant mRNA. Two biological replicates, four samples each (1 control, 3 test samples), representing intact high-quality RNA (50 ng each) were reverse transcribed according to the protocol provided in the Ion AmpliSeq™ Transcriptome Human Gene Expression kit (Revision A.0, Thermo Fisher Scientific). cDNA was amplified using Ion AmpliSeq™ Transcriptome Human Gene Expression core panel (Thermo Fisher Scientific) and primer sequences were partially digested followed by adaptors ligation (Ion P1 Adapter and Ion Xpress™ Barcode Adapter, Thermo Fisher Scientific). Adaptor-ligated amplicons were purified using the Agencourt® AMPure® XP reagent (Beckman Coulter) and eluted into the amplification mix (Platinum® PCR SuperMix High Fidelity and Library Amplification Primer Mix, Thermo Fisher Scientific) and amplified. Size-selection and purification was conducted using Agencourt® AMPure® XP reagent (Beckman Coulter). Amplicons were quantified using the Fragment Analyzer^TM^ instrument (Advanced Analytical Technologies, INC.) with the DNF-474 High Sensitivity NGS Fragment Analysis Kit (Advanced Analytical Technologies, INC.). Samples were then pooled (8 samples per pool) followed by emulsion PCR on the Ion OneTouch™ 2 system with the Ion PI™ Template OT2 200 Kit v3 (Thermo Fisher Scientific) and enrichment using the Ion OneTouch™ ES (Thermo Fisher Scientific). Samples were loaded on an Ion PI™ chip (Kit v3) and sequenced in the Ion Proton™ System using the Ion PI™ Sequencing 200 Kit v3 *(*Thermo Fisher Scientific).

### RNA sequencing analysis

All libraries were aligned to the human genome (hg38) using the Rsubread aligner (version 1.24.2) (Liao et al., 2013). The number of fragments overlapping each Entrez gene were summarized using featureCounts (Liao et al., 2014) and NCBI RefSeq annotation. Gene annotation was downloaded from the NCBI (ftp://ftp.ncbi.nlm.nih.gov/gene/DATA/GENE_INFO/). Differential expression analyses were undertaken using the edgeR (Robinson et al., 2010) and limma (Ritchie et al., 2015) software packages. Any genes which did not achieve a count per million mapped reads (CPM) >1 in at least 2 samples were deemed to be unexpressed and subsequently filtered from the data. Furthermore, genes without current annotation were removed. Compositional differences between libraries were normalized using the TMM method (Robinson and Oshlack, 2010). All counts were then transformed to log_2_-CPM with a prior count of 2. Differential expression was assessed using linear models and robust empirical bayes *t*-statistics with a trended prior variance (Phipson et al., 2016). *P*-values were adjusted to control the false discovery rate (FDR) below 5% using the Benjamini and Hochberg method. Gene ontology and KEGG pathway analyses used the limma package functions goana and kegga. All sequence data generated in this experiment are available at the NCBI Sequence Read Archive (PRJNA471772).

### Quantitative PCR (qPCR)

To verify differential gene transcription in differentiated Caco-2 cells and compare transcriptional responses between differentiated and proliferating cells, qPCR was performed on a group of selected genes from three biological replicates (Materials and methods, host-parasite interaction section). RNA (1 μg/sample) was reverse transcribed to cDNA, using the RevertAid H Minus First Strand cDNA Synthesis Kit (Thermo Fisher Scientific), according to the manufacturer's instructions. The synthesized cDNA (5 ng per well) was subject to qPCR amplification incorporating 1x of Maxima SYBR Green/ROX qPCR Master Mix into the reaction well (Thermo Fisher Scientific) and 250 nM of each forward and reverse primers. The human GAPDH gene was used as an endogenous control. qPCR reactions were run in a StepOne Plus real time PCR machine (Applied Biosystems, Thermo Fisher Scientific, MA, USA) with the following cycling conditions: polymerase activation at 95°C (10 min), 40 cycles of denaturation at 95°C (15 s), annealing at 60°C (30 s), and extension at 72°C (30 s) followed by melt curve analysis as part of the default run settings. Fold change in RNA levels was calculated using the ΔΔCt method.

### Collection of proteins and western blots

Host-parasite interaction experiments were repeated but with two extra time points included (6 and 10h). A slight modification to sample collection was required to eliminate serum contamination. Specifically, we included two extra washes with PBS after trophozoite detachment in which cells were scraped off and pelleted by centrifugation (750 xg, 7 min, 4°C) in the last wash. Cellular pellets (control, and interactions 1.5–10 h) were processed to obtain both cytoplasmic and nuclear fractions following the manufacturer's instructions for the NE-PER™ Nuclear and Cytoplasmic Extraction Reagents (Thermo Scientific) kit. Each extraction reagent was supplemented with Halt™ Protease Inhibitor Cocktail (Thermo Scientific) and phosphatase inhibitors (Pierce, Thermo Scientific) at the concentrations recommended by the suppliers. To prepare a total cell lysate for further studies, parasitized Caco-2 cells and controls were lyzed directly in RIPA buffer (with HALT protease and phosphatase inhibitors), followed by sonication for 30 s (50% amplitude) to reduce samples viscosity. For Western blot analyses, protein samples (30 μg each) were electrophoresed (100 V) on AnykD gels (Biorad, CA, USA) and transferred onto a PVDF membrane for 90 min at 100 V (4°C). Blots were blocked with 5% non-fat dry milk in Tris Buffered Saline with 0.1% Tween-20 (TBST) for 1 h at room temperature (RT) followed by the addition of primary antibodies diluted in either 5% nonfat dry milk or 5% BSA in TBST and incubation overnight (4°C). The following primary antibodies targeting proteins of the MAPK signaling pathway were used: phosphorylated Erk1/2 (p-Erk1/2), phosphorylated P38 (p-P38), phosphorylated SAPK/JNK (p-SAPK/JNK), NFκB p105/50 (for both nuclear and cytoplasmic fractions blots), GAPDH (loading control, Sigma-Aldrich) and TATA box binding protein TBP as a nuclear loading control (Thermo Fisher). Blots prepared with total cell lysates were probed with the Cell Cycle and Apoptosis WB Cocktail (pCdk/pHH3/Actin/PARP) (Abcam, Cambridge, UK) and with the phosphor-H2A.X (ser139) antibody to check for DNA damage (Thermofisher). In the following day, blots were washed three times with TBST (5 min each) and incubated at RT with anti-mouse and anti-rabbit horse radish peroxidase conjugated secondary antibodies (in 5% nonfat milk-TBST), developed using the Clarity™ ECL Western Blotting Substrate (Biorad) and viewed using a ChemiDoc Imaging System (Biorad). For re-probing, membranes were stripped using either a mild (200 mM glycine, 1% SDS and 1% Tween-20, pH 2.2) or harsh (62.5 mM Tris, pH 6.8, 2% SDS, 114 mM β-meracapoethanol) stripping buffer, blocked and re-probed with antibodies against the non-phosphorylated form of MAPK signaling proteins: Erk1/2, P38 and SAPK/JNK. All primary antibodies were used at 1:1000 dilution. GAPDH primary antibody, and anti-mouse and anti-rabbit secondary antibodies were used at a 1:10000 dilution. All antibodies used were purchased from Cell Signaling Technology (MA, USA) unless otherwise stated. The above experiments were repeated three times to verify the results of Western blots. Densitometry for detected bands was performed using the Image J software (https://imagej.nih.gov/ij/, 1997-2016).

### Immunofluorescence

Immunofluorescence was performed on chamber slides (Materials and methods, human intestinal epithelial cells section) to detect the nuclear translocation of NFκB. Before interactions, both IECs grown in wells and trophozoites were washed with warm PBS. Trophozoites were processed as described in a previous section (Materials and methods, host-parasite interactions experiment section) and 4.2 × 10^5^ trophozoites in complete DMEM (HI-FBS) were added to IECs (differentiated or proliferating) and allowed to interact for 1.5, 3, 4.5, 6, and 10 h (37°C, 10% CO_2_). IECs incubated with DMEM media only served as a negative control and those incubated with 100 ng/ml of TNF-α in DMEM as a positive control. After interactions, chamber slides were washed once with warm PBS (37°C) and fixed in 4% paraformaldehyde in PBS (15 min at RT). Following fixation, wells were washed (3x in PBS) and blocked in PBS with 5% normal goat serum and 0.3% triton-X100 for 2 h at RT. Primary antibody (in PBS with 1% BSA and 0.3% triton X-100) targeting the human NFκB (1:200 dilution) was added to wells and the slides were incubated overnight at 4°C. Slides were washed next day (3x in PBS) and probed with an Alexa Fluor™488-conjugated anti-rabbit antibody for 1 h at RT then washed 4 times with PBS and mounted using a Vectashield anti-fade reagent with DAPI (nuclear stain) (Vector Laboratories, CA, USA). Images were taken using an Axioplan 2 fluorescent microscope (Zeiss, oberkochen, Germany).

### AP-1 reporter plasmid assay

To detect AP-1 activation in IECs co-incubated with the GS isolate, Caco-2 cells were transfected with the plasmid pGL4.44[luc2P/AP1 RE/Hygro] expressing the luciferase reporter gene *luc2P* (*Photinus pyralis*) under the control of a promoter that contains the transcription response element (TRE) of AP-1. Transfection of Caco-2 cells was performed as described previously (Ma'ayeh et al., 2017). Trophozoites (5 × 10^4^) were processed (as described in the host-parasite interaction experiment section) and added to transfected cells in a total volume of 75 μl DMEM per well. Trophozoites were allowed to interact with IECs for 1.5, 3, 4.5, 6, and 10 h (37°C, 10% CO_2_) and cells incubated alone in DMEM served as a control. As a positive control, Phorbol 12-myristate 13-acetate (PMA) (Promega), was added to IECs at a 5 μM concentration. After interactions, luminescence reads were assayed using the Dual-Glo® Luciferase reagent following the manufacturer's instruction (Promega). In total, three biological replicates were performed separately to assess AP-1 activation.

### Enzyme linked immunosorbent assay (ELISA)

To measure the cytokines released by differentiated Caco-2 cells, ELISA was performed on the spent media collected from the interaction experiments. The assayed cytokines include the CXCL1, CXCL8 (IL-8), CCL20, IL-1-α, IL-1-β, and TNF-α. Kits used for cytokines measurement were purchased from R&D Systems (MN, Canada) and measurements were performed following the manufacturer's instructions. Absorbance reads from samples and standard curves were plotted into an ELISA analysis software (http://www.elisaanalysis.com), which uses a four-parameter logistic ELISA curve fitting to obtain cytokine concentrations.

### Cytokines degradation by GS isolate secreted proteins

GS trophozoites SPs were collected in a serum-free medium and concentrated as previously described (Ma'ayeh et al., 2017). SPs were then incubated with different cytokines to test for cytokine degradation. The human recombinant interlukin-8 (IL-8), chemokine ligand-20 (CCL20) and tumor necrosis factor-alpha (TNF-α) were purchased from Sigma-Aldrich whereas chemokine ligand 1 and 3 (CXCL1 and CXCL3) and interleukin 1-alpha (IL-1-α) were purchased from R&D Signaling (R&D Systems). All cytokines were suspended in a sterile PBS at a concentration of 100 ng/μl. SPs were quantified for total proteins using the Qubit protein assay reagent (Thermo Fisher Scientific), upon which 1 μg of SPs was mixed with each one of the cytokines above (1 μg) and incubated at 37°C for 1 h. Cytokines or SPs incubated alone served as controls. For IL-8 and CCL20 only, we tested the effect of increasing SPs concentration (1, 5, and 10 μg) on cytokine degradation. After incubations, samples were mixed with Lammelli sample buffer, boiled for 5 min, cooled on ice and loaded into Any kD™ Mini-PROTEAN® TGX Stain-Free™ Protein Gels (Bio-Rad) alongside a Spectra™ Multicolor Broad Range Protein Ladder (Thermo Fisher Scientific). Gels were run at 100 volts until the dye reached the bottom of the gel. Gels were fixed and stained using a QC Colloidal Commassie Stain (Bio-Rad) and visualized using a ChemiDoc Imaging System (BioRad).

### Statistical analysis

The data obtained from AP-1 measurements and ELISAs were analyzed using a one-way analysis of variance (ANOVA) at α < 0.05, followed by Bonferroni comparisons (*P* < 0.05) to identify significant differences between the test groups or test groups versus control. The change in RNA levels between parasitized differentiated and proliferating Caco-2 cells were compared using a two-way ANOVA (α < 0.05) followed by Tukey's pairwise comparisons (*P* < 0.05) to identify significant differences for each gene.

## Results

### Differentially transcribed genes (DTGs) in differentiated Caco-2 cells

RNA Sequencing (RNA Seq) was performed on differentiated Caco-2 cells exposed to trophozoites of the GS isolate for 1.5, 3, and 4.5 h, including a control of differentiated Caco-2 cells incubated alone in DMEM. Between 82 and 84% of each raw read library were successfully mapped to the human genome, and at least 90% of the mapped reads fell within annotated coding domains (12,266 in total), which were transcribed across all experimental Caco-2 cell samples. Overall, compared to control, 220 (Table S1), 1378 (Table S2) and 2500 (Table S3) DTGs were identified at 1.5, 3, and 4.5 h, respectively (Figure S1). The intersection of DTGs at each time point is presented in a Venn diagram (Figure S1A). Additional comparisons showed that 82 (54 up-regulated and 28 down-regulated, Table S4) and 9 (2 up-regulated and 7 down-regulated, Table S5) genes were differentially transcribed when samples from 3 and 4.5 h, respectively, were compared to their previous time point (Figure S1A). Comparisons amongst the transcriptomes at the three different time points also identified 173 overlapping DTGs and others specific to each comparison pair (Figure S1A, Table S6). A sub-set of DTGs were verified with qPCR (for the three time points), and the results correlated well with RNA Seq data (R^2^ = 0.88, 0.85, and 0.91 for 1.5, 3 and 4.5 h, respectively) (Figures S1B, C) (*n* = 28, 3 biological replicates). RNA levels of the same DTGs were also compared between parasitized proliferating and differentiated Caco-2 cells and this showed significant differences between the two groups at each time point (1.5 h *P* = 0.0006, 3 h *P* = 0.0096 and 4.5 h *P* = 0.0015) with a noticeable higher fold change for most of the genes in differentiated IECs (See heat map, Figure S2). Nevertheless, many inflammation-associated genes (e.g., interleukin-8, C-X-C chemokine ligand 1-3 and C-C ligand 20) were up-regulated in proliferating IECs (See heat map Figure S2). Although insignificant (*P* > 0.05, Tukey's pairwise comparisons), RNA levels of some chemokines (*cxcl2* at 3 and 4.5 h and *cxcl3* at 3h and *il8* at 1.5 and 4.5 h) were on par or slightly higher in proliferating compared to differentiated IECs (Figure S2). These results indicate that the transcriptional response to *Giardia* trophozoites varies between differentiated and proliferating Caco-2 cells, however, the differential cell status does not interfere with pathogen recognition and the initiation of an immune response.

### Caco-2 cell transcriptome at different time points vs. control

#### Primary response genes

Primary response genes, which include immediate early and delayed genes, play pivotal roles in a wide range of biological processes including differentiation, proliferation, survival, stress, innate and adaptive immune responses, and glucose metabolism (Fowler et al., 2011; Bahrami and Drabløs, 2016). At 1.5 h, many of the up-regulated genes belong to the primary response group (e.g., *fos, fosb, ier3, ier5, nr4a1-3, egr1, egr2, ets2, mcl1, zfp36, insig1, atf3, klf5*, etc). This group is denominated by transcription factors, prompting us to assess the DTGs for enriched GO terms to verify this. The analysis showed that ~54% (118/220) of DTGs have nuclear location (GO:0005634) and ~20% (43/220) are annotated with transcription factor activity (sequence-specific) (GO:0003700) (Table S7). Among the different transcription factors, we identified an enrichment of the GO terms, activator protein 1 (AP-1) [GO:0035976; transcription factor AP-1 complex (*fos* and *jun*)] and nuclear factor kappa B (NFκB) (GO:0033256; I-kappaB/NF-kappaB complex) (Table S7). Both transcription factors (TFs) are activated in response to different stimuli (including pathogens) and they are induced downstream the mitogen activated protein kinase (MAPK) cascade (Neff et al., 2001; Fujioka et al., 2004), which was also enriched in our DTGs (GO:0000165).

At 3 h, many of the primary response genes identified earlier were still differentially expressed at 3 h with ~ 44% (610/1378) of DTGs showing nuclear location (GO:0005634) and ~8% (115/1378) annotated with transcription factor activity (sequence-specific) (GO:0003700) (Table S8). At 4.5 h, the percentages dropped to 41% (1016/2500) and 7% (173/2500) for nuclear location (GO:0005634) and transcription factor activity (sequence-specific) (GO:0003700), respectively (Table S9). The transcriptional level of *relb* and *fkb1*, the subunits of NFκB, peaked at 3 h and there was an increase in the RNA levels of *fos* and *junb* (subunits of AP-1) (Table 1). Nevertheless, the RNA levels of *relb, fkb1* and *junb* declined at 4.5 h but remained up-regulated compared to the control (Table 1).

**Table 1:** Differentially transcribed genes (DTGs) in the differentiated colon adenocarcinoma cell line, Caco-2, during interaction with *Giardia intestinalis* GS isolate.

**Symbol**	**Gene name**	**Fold change**			**Function**
		**1.5h**	**3h**	**4.5h**	
**INNATE IMMUNE RESPONSES**					
*cxcl3*	C-X-C motif chemokine ligand 3	235.1	18.1	7.2	Chemoattractant, monocytes
*ccl20*	C-C motif chemokine ligand 20	153.4	171.9	91.1	Chemoattractant, lymphocytes and DCs
*cxcl1*	C-X-C motif chemokine ligand 1	121.8	26.7	8.1	Chemoattractant, neutrophils
*cxcl2*	C-X-C motif chemokine ligand 2	46.3	3.9	1.5	Chemoattractant, PMN
*cxcl8*	C-X-C motif chemokine ligand 8	41.3	15.2	8.3	Chemoattractant, neutrophils
*cxcl10*	C-X-C motif chemokine ligand 10	33.4	43.7	28.9	Chemoattractant, monocytes, T cells, NK cells and DCs
*ccl2*	C-C motif chemokine ligand 2	22.6	15.5	6	Chemoattractant, monocytes, memory T cells and DCs
*il1a*	interleukin 1 alpha	16.8	10	3.8	Proliferation of T cells, proliferation and maturation of B cells
*csf1*	colony stimulating factor 1	7.2	7.4	3.9	Proliferation and differentiation of monocytes and macrophages
*nos2*	nitric oxide synthase 2	7.1	14.2	10.3	Production of nitric oxide, a cytotoxic compound to microbes
*il1b*	interleukin 1 beta	6.5	8.7	4.5	Neutrophils recruitment, B and T cells activation
*il10ra*	interleukin 10 receptor subunit alpha	3.8	10	3.4	Binds IL10 with a high affinity
*ptgs2*	prostaglandin-endoperoxide synthase 2	3.6	3.4	2	Production of prostaglandins, anti-inflammatory
*ackr3*	atypical chemokine receptor 3 (CXCR7)	3.4	1.3	0.63	Controls chemokine levels and localization
*cx3cl1*	C-X3-C motif chemokine ligand 1	2.8	2.4	2	Chemoattractant, T cells and monocytes
*ifngr1*	interferon gamma receptor 1	1.9	3	3.2	Receptor for the cytokine interferon gamma
*il12a*	interleukin 12A	2.4	3.9	2.5	Enhances lytic activity of activated T and NK cells, induction of interferon gamma by PMNC
*clcf1*	cardiotrophin like cytokine factor 1	1.8	3.9	6	Cytokine with B-cell stimulating capability
*il34*	interleukin 34	0.81	**0.45**	**0.41**	Promotes the proliferation, survival and differentiation of monocytes and macrophages
*xcl1*	X-C motif chemokine ligand 1	0.7	**0.49**	**0.44**	Chemotactic activity for lymphocytes but not for monocytes or neutrophils
*f2rl1*	F2R like trypsin receptor 1	2.1	4.5	4.9	Modulation of inflammatory responses and regulation of innate and adaptive immunity
*cd55*	CD55 molecule (Cromer blood group)	3.3	9.9	12.2	Inhibits complement activation
*il11*	interleukin 11	1.9	5.7	10.8	Stimulates the proliferation of hematopoietic stem cells and megakaryocyte progenitor cells
*ccrl2*	C-C motif chemokine receptor like 2	2.4	3.2	4.3	Plays a critical role for the development of Th2 responses
*tlr2*	toll like receptor 2	0.8	0.5	**0.43**	Cooperates with TLR1 or TLR6 to mediate the innate immune response to bacterial lipoproteins or lipopeptides
**REGULATION OF NUCLEAR FACTOR KAPPA B (NF**κ**B) ACTIVITY**					
*tnfaip3*	TNF alpha induced protein 3	27	2.3	1.3	Terminates NFκB activity
*nfkbid*	NFKB inhibitor delta	14.6	2.5	2.2	Regulation of NFκB activity
*nfkbiz*	NFKB inhibitor zeta	6.2	1.7	1.4	Regulation of NFκB transcription factor complexes
*nfkbie*	NFKB inhibitor epsilon	3.4	1.5	1	Inhibits NFκB by complexing with and trapping it in the cytoplasm
*relb*	RELB proto-oncogene, NF-kB subunit	3.1	5.5	2.9	Subunit of NFκB transcription factor
*nfkb1*	nuclear factor kappa B subunit 1	1.2	3.8	1.5	Pleiotropic transcription factor
**INDUCTION OF ACTIVATOR PROTEIN-1 (AP-1) TRANSCRIPTION FACTOR**					
*fosb*	FosB proto-oncogene	11.3	3.2	2.6	AP-1 transcription factor subunit
*fos*	Fos proto-oncogene	10.3	13	15.9	AP-1 transcription factor subunit
*junb*	JunB proto-oncogene	6.5	6.8	4.5	AP-1 transcription factor subunit
**REGULATION OF MAPK CASCADE**					
*trib1*	tribbles pseudokinase 1	5.8	4.8	3.9	Regulates activation of MAP kinases
*dusp1*	dual specificity phosphatase 1	9.7	11.6	13.3	Inactivation of MAPKs
*dusp4*	dual specificity phosphatase 4	6.0	9.5	9.9	Inactivation of MAPKs
*dusp5*	dual specificity phosphatase 5	5.9	6.8	9.4	Inactivation of MAPKs
*dusp2*	dual specificity phosphatase 2	3.5	3.7	4.5	Inactivation of MAPKs
*mapk14*	mitogen-activated protein kinase 14	0.9	**0.63**	**0.63**	MAPK signal transduction pathway
**MODULATION OF IMMUNE RESPONSES VIA mRNA DECAY**					
*noct*	nocturnin	7.8	4.2	2.7	Binds poly(A) tails of specific mRNAs leading to their degradation
*zfp36*	ZFP36 ring finger protein	6.6	6.8	8.1	Binds AU-rich element (ARE)-containing mRNAs, inducing decay
*zc3h12a*	zinc finger CCCH-type containing 12A	6.2	3.6	2.9	Acts as an endoribonuclease involved in mRNA decay
**CELL CYCLE REGULATION**					
*btg2*	BTG anti-proliferation factor 2	8.2	2.7	2	Anti-proliferative protein
*gadd45b*	growth arrest and DNA damage inducible beta	7.4	2.3	1.3	Regulation of growth and apoptosis
*gadd45a*	growth arrest and DNA damage inducible alpha	4.9	7.2	3.4	Inhibits entry of cells into S phase
*rgcc*	regulator of cell cycle	3.6	2.7	1.9	Modulates the activity of cell cycle-specific kinases
*cks1b*	CDC28 protein kinase regulatory subunit 1B	**0.3**	1	0.9	Essential for biological function of cyclin dependent kinases
*plk3*	polo like kinase 3	3.5	5.9	6.8	Cell cycle regulation
*cdkn1a*	cyclin dependent kinase inhibitor 1A	0.7	0.8	3.2	Mediates p53/TP53 role as an inhibitor of cellular proliferation in response to DNA damage
*rassf4*	Ras association domain family member 4	0.7	**0.4**	**0.2**	May promote apoptosis and cell cycle arrest
*cdk14*	cyclin dependent kinase 14	0.6	**0.4**	**0.4**	Involved in the control of the eukaryotic cell cycle, whose activity is controlled by an associated cyclin
*fancc*	Fanconi anemia complementation group C	0.8	0.7	**0.5**	DNA repair protein that may operate in a post-replication repair or a cell cycle checkpoint function
**REGULATION OF APOPTOSIS**					
*pim3*	Pim-3 proto-oncogene, serine/threonine kinase	5.1	4.6	3	Anti-apoptotic protein
*bbc3*	BCL2 binding component 3	5.0	5.2	4.9	Essential mediator of p53/TP53-dependent or independent apoptosis
*pmaip1*	phorbol-12-myristate-13-acetate-induced protein 1	4.8	3.5	3.4	Promotes activation of caspases and apoptosis
*phlda1*	pleckstrin homology like domain family A member 1	2.7	8.3	7	Regulation of apoptosis
*aen*	apoptosis enhancing nuclease	2.5	3.4	3.5	Mediates p53-induced apoptosis (DNA damage)
*mcl1*	MCL1, BCL2 family apoptosis regulator	2.2	2.8	3.6	Regulation of apoptosis versus cell survival
*tnfrsf10b*	TNF receptor superfamily member 10b	2.0	3.5	3.4	Activates caspase-8 mediated apoptosis pathway
*tnfsf10*	TNF superfamily member 10	0.8	**0.58**	**0.46**	induces apoptosis
*diablo*	diablo IAP-binding mitochondrial protein	2.8	5.6	4.7	Promotes apoptosis by activating caspases in the cytochrome c/Apaf-1/caspase-9 pathway
**GLUCOSE UPTAKE AND PRODUCTION, TOLERANCE TO GLUCOSE STARVATION**					
*pck1*	phosphoenolpyruvate carboxykinase 1	12.1	10.4	5.6	Produces glucose from lactate
*slc2a6*	solute carrier family 2 member 6	3.0	3.4	2.9	Facilitative glucose transporter
*gsk3a*	glycogen synthase kinase 3 alpha	2.6	3.7	1.8	Negative regulator in the hormonal control of glucose homeostasis
*nuak2*	NUAK family kinase 2	2.5	**0.49**	**0.45**	Tolerance to glucose starvation
*slc2a3*	solute carrier family 2 member 3	1.2	2	5.3	Facilitative glucose transporter that can also mediate the uptake of other monosaccharides across the cell membrane
*g6pc*	glucose-6-phosphatase catalytic subunit	1.3	0.9	**0.3**	Hydrolyzes glucose-6-phosphate to glucose in the endoplasmic reticulum, glucose production
**INTESTINAL EPITHELIAL BARRIER FUNCTION**					
*cldn4*	claudin 4	2.1	2.7	3.1	Plays a major role in tight junction-specific obliteration of the intercellular space
*cldn7*	claudin 7	1	11.6	1.2	Plays a major role in tight junction-specific obliteration of the intercellular space
*cdhr5*	cadherin related family member 5	3.9	5.6	5.9	Controls the packing of microvilli at the apical membrane of epithelial cells.
*muc2*	mucin 2, oligomeric mucus/gel-forming	1.6	7.4	19	Provides a protective, lubricating barrier against infectious agents at mucosal surfaces
*cgnl1*	cingulin like 1	0.7	0.5	**0.2**	Anchoring the apical junctional complex, especially tight junctions, to actin-based cytoskeletons
*mpp5*	membrane palmitoylated protein 5	0.7	**0.5**	**0.5**	Plays a role in tight junctions biogenesis and in the establishment of cell polarity in epithelial cells
*cldn19*	claudin 19	0.9	0.6	**0.5**	Plays a major role in tight junction-specific obliteration of the intercellular space
*amot*	angiomotin	0.7	**0.4**	**0.3**	Plays a central role in tight junction maintenance
**Response to oxidative stress**					
*sod2*	superoxide dismutase 2	2.1	2.4	2.1	Destroys superoxide anion radicals
*gpx2*	glutathione peroxidase 2	1.5	3.6	7.6	Protects from the toxicity of hydroperoxides
*srxn1*	sulfiredoxin 1	1.6	2.1	2.1	Resistance to oxidative stress by reducing cysteine-sulfinic acid formed under exposure to oxidants
*sesn2*	sestrin 2	1.6	1.6	1.1	reduction of oxidized peroxiredoxins
*trpm2*	transient receptor potential cation channel subfamily M member 2	2.8	2.8	4.7	Confers susceptibility to cell death following oxidative stress.
*nfe2l2*	nuclear factor, erythroid 2 like 2	1.9	2.6	2.5	Transcription activator that binds to antioxidant response (ARE) elements in the promoter regions of target genes.
*nox1*	NADPH oxidase 1	1.4	1.2	3.1	Generates superoxide and might conduct H^+^ ions as part of its electron transport mechanism
*noxa1*	NADPH oxidase activator 1	0.9	**0.6**	**0.5**	Functions as an activator of NOX1, a superoxide- producing NADPH oxidase.

*The table shows DTGs associated with different functions and the fold change in their RNA level at 1.5, 3, and 4.5 h of interaction. The function of DTGs is derived from the https://string-db.org. The fold change in RNA level for significantly down-regulated genes are indicated in bold font whereas underlined fold change indicates an insignificant change in RNA level*.

### Activation of NFκB and AP-1transcription factors

**Figure 1:**
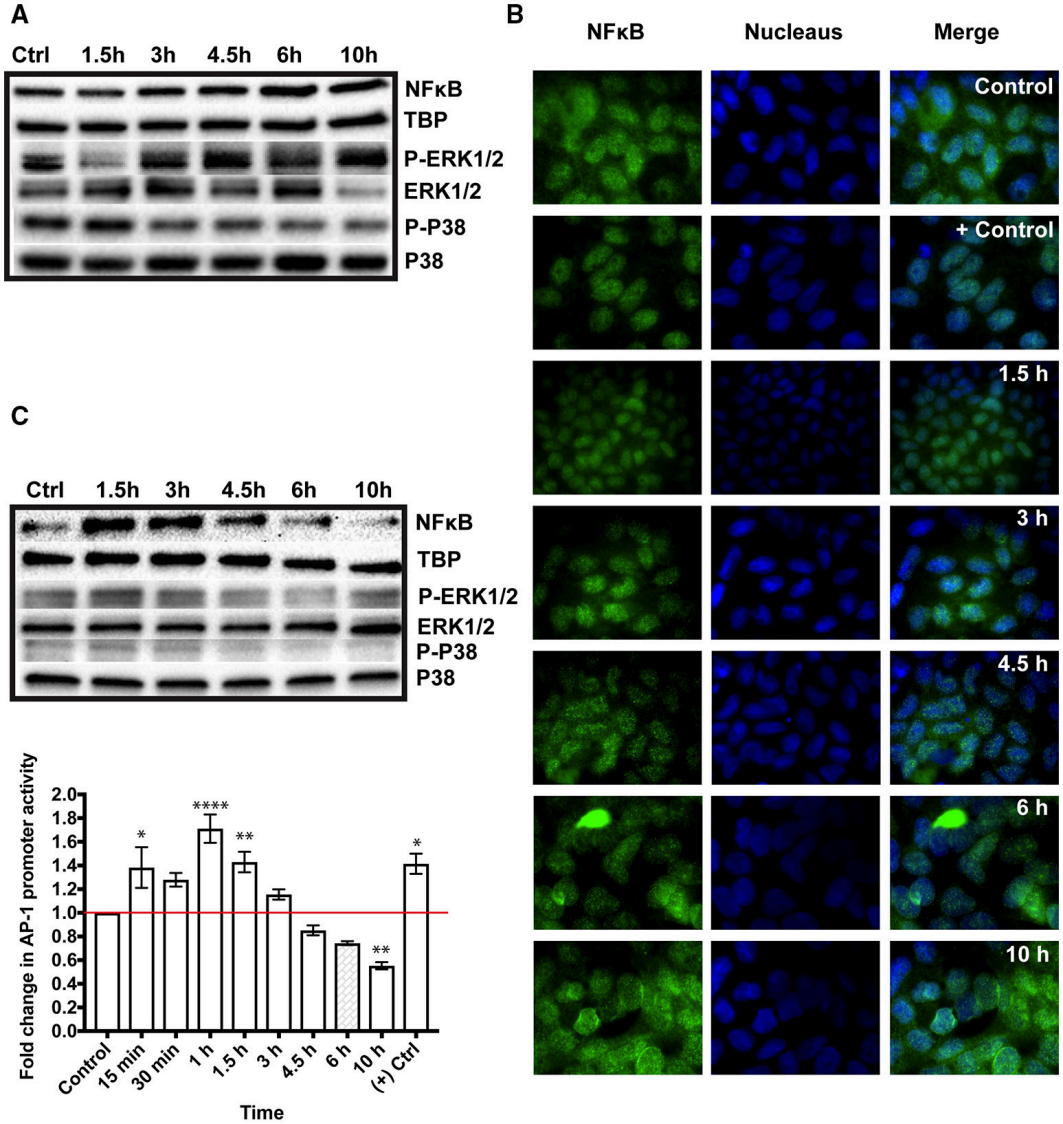
Immune signaling in the colon carcinoma cell line, Caco-2, co-incubated with *Giardia intestinalis* GS isolate (0–10h). The activation of the immune signaling is mediated by the transcription factors, nuclear factor kappa-B (NFκB) and activator protein-1 (AP-1) and phosphorylated mitogen activated protein kinases (MAPKs), ERK1/2 and P38. **(A)** Western blot analysis of NFκB translocation into the nucleus of parasitized differentiated Caco-2 cells. The blots also show the phosphorylation of ERK1/2 (3–10 h) and P38 (1.5 h) in co-incubations with GS trophozoites. **(B)** Immunofluorescent staining of NFκB translocation into the nucleus of parasitized differentiated Caco-2 cells (See fluorescent green dots in the nucleus). Positive control in immunofluorescent images is differentiated Caco-2 cell incubated with 100 ng of tumor necrosis factor alpha per ml of culture medium. Negative control in differentiated Caco-2 cells incubated alone in culture medium. **(C)** Western blot analysis showing the nuclear translocation of NFκB and the activation of AP-1 as assessed by a luciferase reporter system transfected into proliferating Caco-2 cells. Western blots also show a slight increase in Erk1/2 phosphorylation at 1.5 h in proliferating Caco-2 cells co-incubated with GS trophozoites (0–10 h). TATA box binding protein (TBP) is the nuclear loading control used in Western blots. P-Erk is the phosphorylated form of Erk and P-P38 is the phosphorylated form of P38. ^*^P < 0.05, ^**^P < 0.01, and ^****^P < 0.001.

To verify the activation of NFκB in response to GS trophozoites, we produced Western blots with nuclear fractions from differentiated IECs and probed with antibodies against the p50 subunit of NFκB. We detected NFκB in both the control and test samples. At 1.5 h, NFκB band looked slightly fainter than the control (0.84-fold decrease) but higher band intensities were observed thereafter (1.11-, 1.45-, 1.51-, and 1.26-fold increase for 3, 4.5, 6, and 10 h, respectively) indicating the recruitment of more NFκB into the nucleus (Figure 1A) and we confirmed these results further using immunofluorescence imaging (Figure 1B, see fluorescent dots in the nucleus). An image with higher magnification showing NFκB in the nucleus is presented in Figure S3. We then checked for a similar effect in proliferating IECs and followed the kinetics of NFκB activation over time. A strong 50 kDa band was detected at 1.5 h of interaction (2.3-fold increase) declining afterward (2-, 1.5-, 1.1-, and 0.49-fold successively) indicating a peak in NFκB nuclear recruitment at this time point (Figure 1C). These results were also confirmed using immunofluorescent staining (Figure S4). This suggests that NFκB activation in proliferating Caco-2 cells occurs faster than in differentiated cells. Then, we verified the activation of AP-1 in parasitized Caco-2 cells transiently transfected with a luciferase reporter plasmid. We could only test for AP-1 activation in proliferating Caco-2 cells because the plasmid is not retained in differentiated IECs. We increased the resolution of this experiment by including three extra time points (e.g., 15, 30 min and 1 h) to probe the kinetics of AP-1 induction in Caco-2 cells upon addition of GS trophozoites. A significant increase in AP-1 activity was detected as early as 15 min upon adding trophozoites (1.382 ± 0.72-fold increase, *P* < 0.05), peaking at 1 h (1.711 ± 0.12-fold increase, *P* < 0.0001) (Figure 1C). After 1 h, the activity of AP-1 started declining, but remained significant at 1.5 h reaching the lowest by 10 h (0.551 ± 0.031-fold decrease, *P* < 0.01) (Figure 1B). These results demonstrate that co-incubation with GS trophozoites activates AP-1 in Caco-2 cells during the early hours of interaction, a result paralleling that for NFκB nuclear recruitment. Thus, both TFs are activated upon exposure to GS trophozoites.

### Mitogen activated protein kinase signaling

The MAPK signaling pathway includes a group of kinases that function upstream of AP-1 and NFκB leading to their activation (Neff et al., 2001). To test whether co-incubation with GS isolate activated (i.e. phosphorylated) the MAPKs, we prepared Western blots with the cytoplasmic fractions of parasitized Caco-2 cells in both differentiated and proliferating states. The blots were probed with antibodies against the phosphorylated and non-phosphorylated forms of ERK1/2, P38 and JNK. In differentiated Caco-2 cells, we noticed a decreased P-ERK1/2 at 1.5 h (0.53-fold decrease) followed by an increase in phosphorylation peaking at 10 h (1.8-fold, Figure 1A). Notably, this pattern of ERK1/2 phosphorylation was similar to that of NFκB nuclear translocation (Figure 1A). For P38, a phosphorylation signal was only observed at 1.5 h (1.7-fold) followed by a decrease in band intensities thereafter (0.5-fold decrease at 10 h) (Figure 1A), indicating that P38 phosphorylation is being attenuated during co-incubation. For proliferating Caco-2 cells, a slight increase in ERK1/2 phosphorylation was only observed at 1.5 h (1.4-fold) decreasing afterward but reaching a level similar to control at 10 h (Figure 1C). The phosphorylation level of P38 increased slightly at 1.5 and 3 h but decreased thereafter (< 0.7-fold decrease) (Figure 1C). Collectively, these results show that exposure to GS trophozoites induces ERK1/2 phosphorylation in Caco-2 cells but modulates P38 phosphorylation. The results also show that MAPK signaling varies between proliferating and differentiated Caco-2 cells. The phosphorylated form of JNK could not be detected in either cell state.

### Inflammatory responses

NFκB and AP-1 cooperate to induce the transcription of pro-inflammatory genes. We therefore examined the transcriptomes in all time points for the differential transcription of cytokines. In fact, the top up-regulated genes were those encoding the C-X-C motif chemokine ligands 1-3 (*cxcl1-3*) (Table 1). The differential expression of these cytokines was previously seen in Caco-2 cells after co-incubation with WB trophozoites (Roxström-Lindquist et al., 2005) but here we identified a broader range of pro-inflammatory cytokines, specifically the C-X-C motif chemokine ligands 8 (*cxcl8*) and 10 (*cxcl10*), the interleukins 1 alpha (*il1a*) and 1-beta (*il1b*), colony stimulating factor 1 (*csf1*) and C-X3-C motif ligand 1 (*cx3cl1*) (Table 1). The RNA level of genes encoding these pro-inflammatory cytokines peaked at 1.5 h, except for *ccl20, cxcl10, csf1* and *il1b* whose RNA levels peaked at 3 h of interaction (Table 1). The induced genes were enriched for the KEGG pathways ‘cytokine-cytokine interaction pathway’ (hsa04060), ‘TNF signaling pathway’ (hsa04668) and IL17 signaling pathway (hsa04657). Further, the 3 h time point marked the induction of a second group of inflammatory genes which were further up-regulated at 4.5 h. This group includes interleukin 12A (*il12a*), cardiotrophin like cytokine factor 1 (*clcf1*), interleukin 11 (*il11*) and C-C motif chemokine receptor like 2 (*ccrl2*) (Table 1). In addition, we identified a DTG, *nos2*, encoding the enzyme nitric oxide synthase 2 (NOS2). The RNA levels of this gene peaked at 3 h of interaction (Table 1). NOS2 is involved in the host innate immune responses, specifically the production of nitric oxide, a compound cytotoxic to *Giardia* (Roxström-Lindquist et al., 2006). At 4.5 h, most of the aforementioned inflammatory genes, except for *ccl2* and atypical chemokine receptor 3 (*cxcr7*), remained differentially transcribed albeit to a lesser extent (Table 1). Concurrently, other inflammatory genes such interleukin 34 and X-C motif chemokine ligand 1 were down-regulated (Table 1). Therefore, despite the induction and persistence of transcripts encoding inflammatory genes, it appears that by 4.5 h of interaction, a program of anti-inflammatory signals had begun (See Table 1 for target immune cells).

Despite the induction of pro-inflammatory responses, we identified the differential expression of genes associated with different mechanisms regulating inflammation. First, dual specificity phosphatases 1, 2, 4, and 5 were up-regulated (peaking at 4.5 h, Table 1) and mitogen activated protein kinase 14 was down-regulated (3 and 4.5 h, Table 1) in parasitized IECs. These enzymes inhibit MAPKs, attenuating the inflammatory signaling leading to NFκB and AP-1 activation. Second, we identified potential indicators of mRNA decay, which might regulate the levels of cytokine transcripts post-transcriptionally. Specifically, nocturnin (*noct*), zinc finger CCCH-type containing 12 A (*zc3h12a*) and zinc finger protein 36 (*zfp36*) were up-regulated (Table 1), with the latter being among the top 50 up-regulated DTGs at all time points. Third, we identified upregulation of genes associated with inhibition of complement system activation (CD55, complement decay-accelerating factor) (Ozen et al., 2017), regulating chemokine levels and localization (atypical chemokine receptor 3) (Boldajipour et al., 2008; Naumann et al., 2010) and innate and adaptive immunity (F2R like receptor 1) (Shpacovitch et al., 2008) (Table 1). Together, these results suggest the induction of multiple mechanisms for regulating inflammatory responses within IECs upon exposure to GS trophozoites, requiring further experimental verification.

### Cytokine measurements

**Figure 2:**
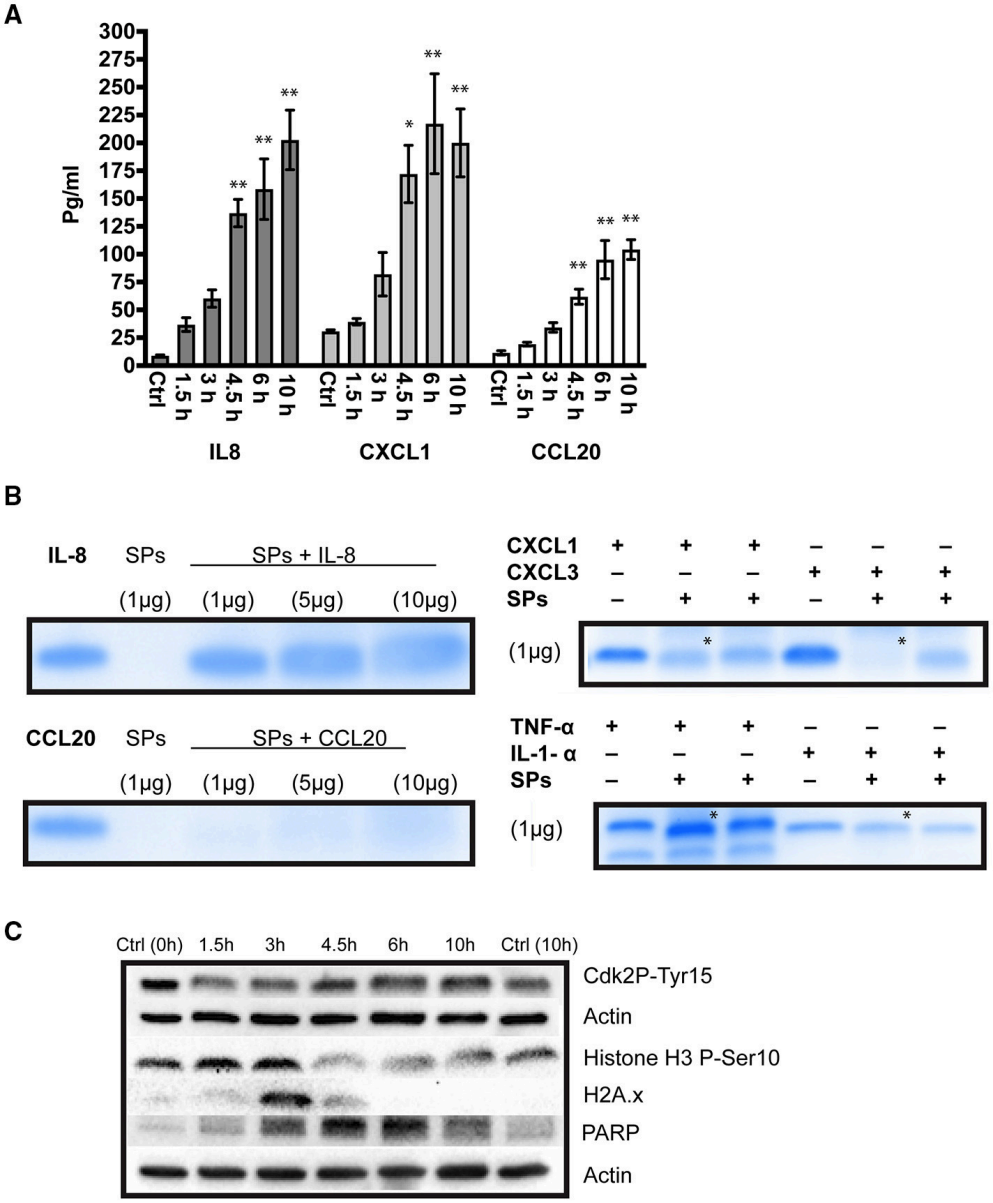
**(A)** ELISA measurements for the cytokines, interleukin 8 (IL-8), C-X-C chemokine ligand 1 (CXCL-1) and C-C chemokine ligand 20 (CCL20), released into the interaction medium of the colon carcinoma cell line, Caco-2, co-incubated with *Giardia intestinalis* GS isolate (0–10 h) ^**^P < 0.01. **(B)** The degradation/cleavage of the cytokines IL-8, CCL20, CXCL1, CXCL3, IL1-α and TNF-α by GS isolate secreted proteins (SPs). The (^*^) on the lanes shows results for the degradation of CXCL1, CXCL3, IL1-α, and TNF-α by SPs from WB isolate (assemblage A), which were included for comparison between assemblages. Note the differences in cytokine degradation for CXCL1, CXCL3, and TNF-α **(C)** Western blot analyses for the changes in cell cycle and induction of apoptosis in parasitized Caco-2 cell (0–10 h). Total cell lysates probed with antibodies to detect cell cycle arrest (phosphorylation of cyclin dependent kinase 2 at Ser15, Cdk2 pTyr15, and histone H3 at Ser10, HH3 pSer10), DNA damage (phosphorylation of histone 2A at Ser139, H2AX) and induction of apoptosis (cleaved Poly [ADP-ribose] polymerase 1, PARP). Note than actin was used as a loading control.

The genes encoding different cytokines were up-regulated in differentiated Caco-2 cells in response to GS isolate infection. The cytokines IL8, CXCL1, and CCL20 play important roles during the early phase of infection to attract different populations of immune cells and thus these were selected for measurements by ELISA. Our aim was to verify whether transcriptional up-regulation correlated with the release of gene products into the interaction media. The amounts of measured cytokines are presented in Figure 2A. Compared to control, the amounts of all measured cytokines increased significantly at 4.5, 6, and 10h of infection (*P* < 0.05) with the highest amounts measured at 10 h of interaction. Compared to their controls, the measured concentrations were higher by ~23-fold for IL-8, ~6.5-fold for CXCL1 and 9.1-fold for CCL20) (Figure 2A). Despite the transcriptional up-regulation of genes encoding TNFα, IL1α and IL1β (Table 1), these cytokines could not be detected in the interaction media. The inconsistency between the amounts of measured cytokines and fold up-regulation in RNA levels (Table 1) together with the inability to detect any TNFα, IL1α, and IL1β in the interaction media suggest a post-transcriptional regulation of their mRNA, implying that the transcripts were either degraded or translationally repressed, or their protein products were degraded in the interaction medium.

### Degradation of cytokines by *Giardia* ESPs

To investigate whether the low levels of measured cytokines was due to degradation, we incubated GS isolate SPs with different cytokines and performed PAGE on the interaction mixture. Incubation of IL-8, CXCL1, CXCL3, and IL1α with GS SPs resulted in the appearance of fuzzy bands on the gels, indicating their cleavage/partial degradation (Figure 2B). The effect of SPs on IL-8 degradation manifested clearly upon incubation with increasing amounts of SPs, where fainter and fuzzier bands could be seen (Figure 2B). CCL20, on the other hand, disappeared completely from the gel upon incubation with 1 μg of parasite SPs, indicating its total degradation (Figure 2B). By contrast, TNFα, did not show any degradation or cleavage upon incubation with GS SPs suggesting a post-transcriptional modification (mRNA degradation or translational repression). Overall, these results demonstrate the SPs of GS isolate selectively degrade host cell cytokines.

### Response to oxidative stress

The production of reactive oxygen species (ROS) by host cells represents a first line of defense against intestinal pathogens (Roxström-Lindquist et al., 2006). ROS are harmful to all cells, and thus the expression of antioxidant enzymes is required to avert cellular toxicity. At 1.5 h, the transcriptome of IECs showed the induction of nuclear factor, erythroid 2 like 2 (*nfe2l2*) together with superoxide dismutase (*sod2*) (Table 1); their RNA levels peaked at 3 h of infection. NFE2L2 is a transcriptional activator that binds the antioxidant response element in the promoter region of target genes initiating transcription (Huang et al., 2000) whereas SOD2 is a canonical eukaryotic ROS detoxification enzyme. The 3 h time point marked the induction of another group of genes whose functions are associated with hydroperoxide detoxification (glutathione peroxidase 2, *gpx2*) (Brigelius-Flohé and Maiorino, 2013), biochemical reduction of cysteine sulfonic acids formed under oxidative stress (sulfiredoxin 1, *srxn1*) (Biswas et al., 2006) and reduction of oxidized peroxiredoxins (sestrin2, *sesn2*) (Essler et al., 2009)(Table 1). The above DTGs remained up-regulated at 4.5 h, especially *gpx2* which exhibited higher RNA levels (Table 1). Despite the induction of antioxidative stress responses, a gene encoding NADPH oxidase 1 (*nox1*), which generates superoxide, was induced at 4.5 h possibly to counteract trophozoites persisting trophozoites on IECs surface (Table 1). The change in RNA levels of the genes above were also presented in a heat map to show temporal changes in responses to oxidative stress in IECs during co-incubation with GS trophozoites (Figure S5). Overall, the results above suggest that IECs produce ROS in response to GS trophozoites but they also up-regulate genes involved in protecting IECs from ROS-induced oxidative damage.

### Cell cycle arrest and apoptosis

Cell death or the regulation of cell death were amongst the highest enriched GO terms in our analysis (GO:0008219 and GO:0010941, respectively) (Tables S7–S9), including other GO terms indicating the induction of the extrinsic apoptotic pathway (Tables S8, S9). These GO terms are death receptor activity (GO:0005035), tumor necrosis factor-activated receptor activity (GO:0005031) and TRAIL binding (GO:0045569). There also existed DTGs encoding mediators of apoptosis via the intrinsic apoptotic pathway, including caspase 9 (*casp9*) and DIABLO (Table 1). Despite the induction of apoptotic DTGs (*bbc3, pmaip1, aen, phlda1, tnfrf10b* and *diablo*), anti-apoptotic genes were differentially transcribed (up-regulation of *pim3*, downregulation of *tnfsf10 and casp6*) (Table 1). Whether parasitized IECs undergo apoptosis is difficult to conclude based on the transcriptome alone, hence, we tested the cleavage of Poly [ADP-ribose] polymerase 1 (i.e., PARP), as a marker of apoptosis. A band of 89 kDa representing cleaved PARP was present in all interaction samples (Figure 2C), indicating the induction of apoptosis in IECs in response to GS isolate trophozoites.

The induction of apoptosis in IECs by *Giardia* has been previously ascribed to stress such as nutrients depletion (e.g., glucose and arginine) or oxidative stress (Roxström-Lindquist et al., 2005; Yu et al., 2008; Stadelmann et al., 2012). In line with this, the IECs transcriptome indicated glucose starvation as a potential stressor. Specifically, the induction of genes associated with tolerance to glucose starvation (*nuak2*), facilitative glucose transport (*slc2a6* and *slc2a3*) and the conversion of lactate (*pck1*) or glucose 6-phosphate to glucose (*g6pc*) (Table 1). Amongst the DTGs, *nuak2* was up-regulated at 1.5 h but down-regulated at later time points (Table 1), indicating that IECs may become less tolerant to glucose starvation over time.

Oxidative stress induces DNA damage, which if not fixed, leads to cell cycle arrest and apoptosis (Clopton and Saltman, 1995). In fact, the cell cycle-associated genes encoding BTG anti-proliferation factor 2 (*btg2*), growth arrest and DNA damage inducible beta (*gadd45b*), regulator of cell cycle (*rgcc*), CDC28 protein kinase regulatory subunit 1B (*cks1b*) and cell division cycle 23 (*cdc23*) were amongst the highest DTGs at 1.5 h (Table 1). The 3 and 4.5 h time points marked the up-regulation of additional genes associated with the cell cycle, including DNA damage inducible alpha (*gadd45a*), an inhibitor of cell entry into the S phase (Smith et al., 1994), and a cyclin dependent kinase inhibitor 1A (*cdkn1a*), an inhibitor of cellular proliferation in response to DNA damage (Bunz et al., 1998) (Table 1). Cell cycle associated genes were presented in a heat map to show temporal changes in their RNA levels during co-incubation with GS trophozoites (Figure S5). To gain some insights on the cell cycle state of parasitized differentiated IECs, total cell lysates were probed with an antibody that detects the phosphorylated form of histone 2A at Ser139 (H2AX), a repair protein recruited to the sites of DNA damage. H2AX was phosphorylated earlier during infection (1.5–4.5 h), indicating DNA damage during this period (Figure 2C). We also tested for cell cycle arrest by probing for cyclin dependent kinase 2 (phosphorylation at Ser15, Cdk2 pTyr15) and histone H3 (phosphorylation at Ser10, HH3 pSer10). HH3 phosphorylation was pronounced at 1.5 and 3 h whereas Cdk2 phosphorylation was seen at later time points (compare to 10 h control, Figure 2C), indicating cell cycle arrest in G1/S phase or M phase, respectively. These results show that IECs exposure to GS trophozoites arrests the cell cycle, an effect mediated, at least in part, by DNA damage.

### Intestinal epithelial barrier function

*Giardia* infection delocalizes and degrades intercellular junctions (e.g., tight junctions and adherens junctions), affecting the permeability of the intestinal epithelial barrier (Troeger et al., 2007; Humen et al., 2011; Halliez et al., 2016). The genes encoding the tight junction proteins claudin 4 (*cldn4*) (all time points) and claudin 7 (*cldn7*) (only at 3 h) were up-regulated (Table 1), and this response is usually interpreted as a compensation mechanism for their loss/degradation by trophozoites. Likewise, the gene associated with the packing of microvilli at the apical membrane of epithelial cells (cadherin related family member 5, *cdhr5*) and the gene encoding mucin 2 (*muc2*) a major component of the mucus layer covering enterocytes were also up-regulated (Table 1). The parasitized IECs transcriptome further indicated effects on tight junctions' biogenesis and integrity as well as cells polarity. These effects are represented by the down-regulation of the genes angiomotin (*amot*), responsible for tight junction maintenance (Wells et al., 2006) and membrane palmitoylated protein 5 (*mpp5*), required for tight junction biogenesis and establishing cell polarity (Roh et al., 2003) (Table 1). Therefore, the changes in transcription of the above genes in parasitized IECs indicate an overall effect on the tight junctions, microvilli and the mucus layer at the enterocyte surface.

### Comparing transcriptomes at consecutive time points

The transcriptome of parasitized IECs at 3 h was compared to that at 1.5 h to specify significant and temporal changes in gene transcription for the period in between (Table S4). At first, we looked at DTGs for the enrichment of certain biological functions, which highlighted a down-regulation in the GO terms chemotaxis for neutrophils and granulocytes, respectively (GO:0090023 and GO:0071624) and in chemokine-mediated signaling pathway (GO:0070098). This more likely related to the down-regulation of *cxcl1*-*3* and *cxcl8* (Table S10). Despite the down-regulation of these GO terms, *nfkb1*, was up-regulated and its inhibitors were down-regulated (*nfkbie* and *nfkbiz*) (Table 2), indicating further activation of NFκB, which is consentient with the results of Western blot analysis (Figure 1A).

**Table 2:** Differentially transcribed genes (DTGs) in the differentiated colon adenocarcinoma cell line, Caco-2, during interaction with *Giardia intestinalis* GS isolate.

**Gene symbol**	**Gene name**	**Fold change**	**Function**
**CELL CYCLE**			
*cks1b*	CDC28 protein kinase regulatory subunit 1B	2.98	Binds to the catalytic subunit of the cyclin dependent kinases and is essential for their biological function
*cdc73*	Cell division cycle 73	2.76	Involved in cell cycle progression through the regulation of cyclin D1/PRAD1 expression
*wee1*	WEE1 homolog (S. pombe)	2.6	Negative regulator of entry into mitosis (G2 to M transition)
*sat1*	Spermidine/spermine N1-acetyltransferase 1	2.12	Attenuation of the intracellular concentration of polyamines. Also involved in the regulation of polyamine transport out of cells, regulation of cell proliferation
*hbp1*	HMG-box transcription factor 1	1.85	Regulation of the cell cycle, cell cycle arrest
*gadd45b*	Growth arrest and DNA-damage-inducible, beta	0.32	Regulation of growth and apoptosis
*btg2*	BTG family, member 2	0.3	Anti-proliferative protein
*sik1*	Salt-inducible kinase 1	0.24	Cell cycle regulation, gluconeogenesis and lipogenesis
*gadd45g*	Growth arrest and DNA-damage-inducible, gamma	0.13	Regulation of growth and apoptosis
**TLR SIGNALING AND IMMUNE RESPONSE**			
*nfkb1*	Nuclear factor of kappa light polypeptide gene enhancer in B-cells 1	3.3	Control of inflammation, immunity, differentiation, cell growth, tumorigenesis and apoptosis
*tnfaip3*	Tumor necrosis factor, alpha-induced protein 3	0.86	Immune and inflammatory responses signaled by cytokines or Toll-like receptors (TLRs) through terminating NF-kappa-B activity
*nfkbie*	Nuclear factor of kappa light polypeptide gene enhancer in B-cells inhibitor, epsilon	0.45	Inhibits NF-kappa-B by complexing with and trapping it in the cytoplasm
*fosb*	FBJ murine osteosarcoma viral oncogene homolog B	0.28	Subunit of AP-1 transcription factor
*nfkbiz*	Nuclear factor of kappa light polypeptide gene enhancer in B-cells inhibitor, zeta	0.27	Inhibits NF-kappa-B activity without affecting its nuclear translocation upon stimulation
**TRANSPORT**			
*slc38a2*	Solute carrier family 38, member 2	13.9	Co-transport of neutral amino acids and sodium ions
*slc7a11*	Solute carrier family 7 (anionic amino acid transporter light chain, xc- system), member 11	3.47	Sodium-independent, high-affinity exchange of anionic amino acids with high specificity for cystine and glutamate
*slco2a1*	Solute carrier organic anion transporter family, member 2A1	2.4	Mediates the release of newly synthesized prostaglandins from cells, and the transepithelial transport of prostaglandins
**LIPID METABOLISM**			
*ldlr*	Low density lipoprotein receptor	2.86	Binds LDL, the major cholesterol-carrying lipoprotein of plasma, and transports it into cells by endocytosis
*lpin2*	Lipin 2	2.07	Controls the metabolism of fatty acids at different levels
*agpat9*	1-acylglycerol-3-phosphate O-acyltransferase 9	1.89	Transfer of acyl group from acyl-coA to position sn-1 or sn-2 to glycerol-3-phosphate or 1-acyl-sn- glycerol-3-phosphate to synthesize glycerolipid or lysophosphatidic acid, respectively
**APOPTOSIS**			
*phlda1*	Pleckstrin homology-like domain, family A, member 1	3.04	Regulation of apoptosis
*tnfrsf21*	Tumor necrosis factor receptor superfamily, member 21	2.04	Promotes apoptosis, possibly via a pathway that involves the activation of NF-kappa-B

*The Table shows a group of DTGs with specific functions that were identified by comparing the 3h to the 1.5 h transcriptomes of parasitized IECs. The function of DTGs is derived from the https://string-db.org*.

The above period also marked transcriptional changes associated with a dysregulation of cell cycle. Specifically, the down-regulation of genes involved in cell cycle regulation (e.g., *gadd45b, gadd45g, btg2* and *sik1*) and the up-regulation of others associated with cyclins function (*cks1b* and *cdc73*) or cell cycle arrest (*wee1, hpb1* and *sat1*) (Table 2). Amongst the different DTGs, Wee1-like protein kinase (wee1) and HMG-Box containing protein 1(*hpb1*) was previously shown to be involved in preventing cells entry into mitosis (Harvey and Kellogg, 2003) and cell cycle arrest (Tevosian et al., 1997), respectively. Interestingly, the overall outcome of these changes at gene level could be seen at cellular levels as shown in our western blot analysis (Figure 2C).

In this comparison, we further identified DTGs associated with transport functions, specifically amino acids. An example is the solute carrier family 38 member 2 (*slc38a2*) which functions in the co-transport of amino acids and sodium ions and a non-sodium dependent transporter of amino acid with a specificity for cystine and glutamate was up-regulated (Solute carrier family 7 member 11) (Table 2). Genes associated with cholesterol transport into the cells (low density lipoproteins receptor), and fatty acids metabolism (lipin 2 and 1-acylglycerol-3-phosphate O-acyltransferase 9) were also up-regulated (Table 2). The overall changes in the RNA levels of the genes above may suggest a mechanism to out-compete GS trophozoites for the acquisition of amino acids, cholesterol and lipids.

The transcriptome of parasitized Caco2 cells at 4.5 h was also compared to that at 3 h to identify DTGs in this period, if any. Only eight DTGs were identified and their functions were unrelated (Table S5). Amongst the eight genes, three gene were down-regulated and their functions are associated with transcription [Scaffold attachment factor B (safb)] (Garee and Oesterreich, 2010) and polymerase (DNA-directed) gamma 2 (polg2), and chromatin structure [H1 histone family member x (h1fx)] (Happel and Doenecke, 2009) (Table S5). Therefore, this period of co-incubation with GS trophozoites appears to mark important changes in transcription and chromatin structure.

## Discussion

*Giardia intestinalis* GS isolate (assemblage B) was originally isolated from a patient with severe diarrhea (Nash and Keister, 1985) and has since been extensively used in both *in vivo* and *in vitro* studies to investigate virulence and pathogenesis. Collectively, previous studies showed that the pathologies associated with GS isolate infection involve changes in cell structure and physiology as well as the function of the intestinal epithelial barrier (Humen et al., 2011; Bénéré et al., 2012). However, information on molecular changes in IECs are incomplete and thus we aimed to identify transcriptional and cellular responses during interaction with this isolate.

RNA sequencing was performed on differentiated IECs infected with GS isolate for short periods of time (1.5, 3 and 4.5 h) during which transcriptional changes were identified. A dominance of immediate early response (IER) genes was seen at 1.5 and 3 h and this response is not uncommon, since many stimuli are known to rapidly but transiently induce many IER genes in different cell types (Fowler et al., 2011). An over-representation of TFs binding sites (e.g., NFκB, serum-response factor and cyclic AMP response element binding protein) exists in IER genes (Bahrami and Drabløs, 2016), amongst which NFκB was enriched in our analysis. We confirmed NFκB nuclear translocation during interaction and have previously shown NFκB induction, together with AP-1 (Figure 1), in response to GS isolate SPs (Ma'ayeh et al., 2017). NFκB is a master regulator of innate immune responses, and functions to counteract ROS and promote cell survival (Vallabhapurapu and Karin, 2009).

The MAPK cascade plays an important role in mediating cellular signaling and the activation of NFκB and AP-1 leading to transcription of target genes (Neff et al., 2001). So far, there have been no reports of MAPKs phosphorylation in response to *Giardia* trophozoites, however, a previous study showed that both ERK1/2 and P38 were phosphorylated in HT-29 cells exposed to 1 μg of GS isolate SPs (Lee et al., 2012). Here, ERK1/2 was phosphorylated during interaction with GS trophozoites (Figure 1) whereas the phosphorylation of P38 was attenuated. This indicates that the co-incubation of parasite with IECs modulates P38 phosphorylation, which is an interesting question for future research.

Co-incubation with the GS isolate induced the transcription of genes encoding inflammatory molecules (e.g., cytokines, chemokines and interleukins) in IECs. By comparison, a previous microarray study showed that WB isolate induced the transcription of *cxcl1-3, ccl20* and *ccl2* but not genes like *il8, il1a* or *ilb* (Roxström-Lindquist et al., 2005). Interestingly, there is an accumulating experimental evidence showing that assemblage B, including GS isolate, produces more inflammatory responses both *in vitro* and *in vivo* compared to trophozoites that belong to assemblage A. For example, human peripheral mononuclear cells and the human monocytic cell line (THP-1) produced more TNF-α, IL1-B, INF-γ, and IL-8 during *in vitro* exposure to GS isolate compared to WB (Lee et al., 2012). *In vivo*, some patients with chronic assemblage B infection develop microscopic duodenal inflammation (Hanevik et al., 2007) and experimental infections with assemblage B in mice produced greater polymorphonuclear leukocytes infiltration in the ileum, and robust intestinal inflammatory responses in gerbils (Bénéré et al., 2012). Together, these findings suggest that assemblage B isolates, including GS, have the potential to induce inflammatory responses within the host.

**Figure 3:**
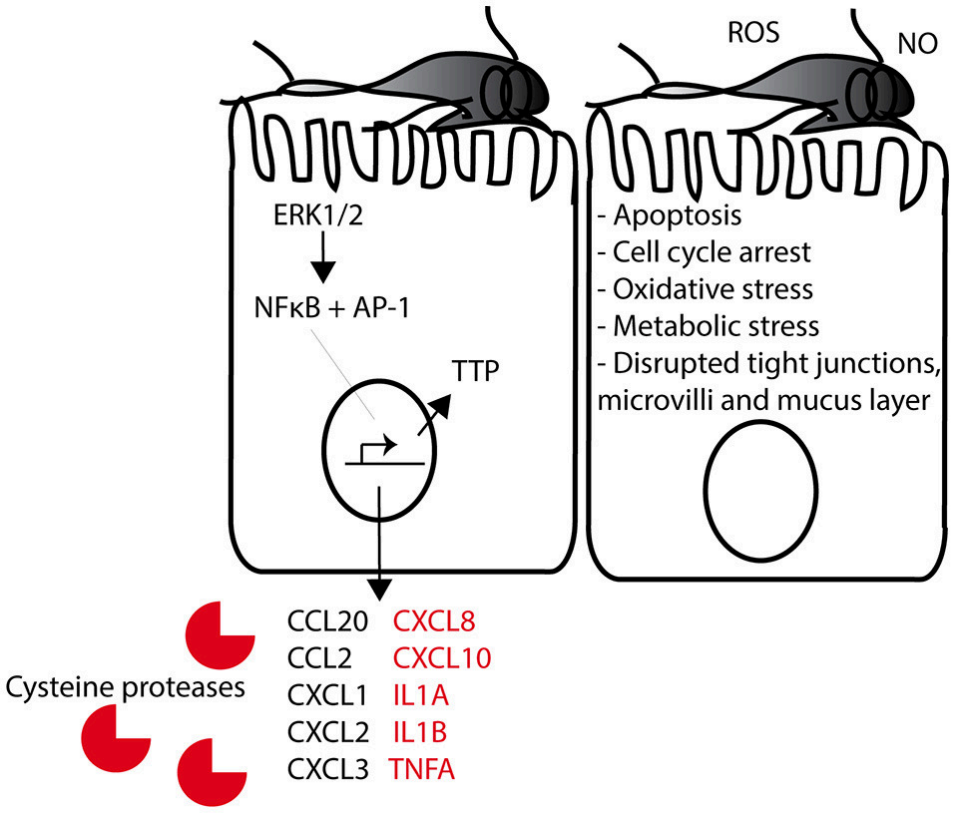
Suggested model of GS trophozoites interaction with intestinal epithelial cells (IECs). *Giardia* trophozoites bind to the IECs with their adhesive discs. Early interaction induces a change in ERK1/2 signaling (i.e. phosphorylation), resulting in the translocation of the transcription factors NFκB and AP-1 into the nucleus. These two transcription factors induce the transcription of a large number of genes, among these several cytokines that can attract immune cells to the intestinal mucosa during infection. Cytokines in red are up-regulated by GS trophozoites (assemblage B) but not by WB trophozoites (assemblage A). The RNA binding protein TTP is induced later during interaction and this will result in the degradation of chemokine/cytokine mRNAs. Reactive oxygen species (ROS) and nitric oxide (NO) is produced by the IECs as part of the innate immune response to *Giardia*. The host-parasite interaction induces apoptosis, cell cycle arrest, oxidative stress, metabolic stress, and disrupted tight junctions and mucus layer on the IECs.

IECs released IL-8, CXCL1 and CCL20 upon incubation with GS trophozoites. Their amounts, however, did not correlate with the strong induction observed in the transcriptome (Table 1). Furthermore, despite the up-regulation of genes such as *il1a, il1b* and *tnfa*, we could not detect these cytokines in the interaction medium. These findings suggest that the mRNAs of the above genes were either not translated (e.g., mRNA degradation) or their protein counterparts were degraded upon release into the medium. In fact, we showed that GS isolate SPs degraded IL-8, IL1-α, CCL20, CXCL1, and CCL20, an effect similar to that of WB isolate (Liu et al., 2018), supporting the notion that these inflammatory cytokines are degraded upon release from IECs. This effect has been specifically ascribed to the cysteine proteases released by the parasite (Cotton et al, 2014; Liu et al., 2018), which has been also shown to attenuate IL-8-induced chemotaxis of neutrophils *in vitro* (Cotton et al, 2014). TNF-α, on the other hand, could not be degraded/cleaved by parasite SPs and thus it is possible that its mRNA was degraded (i.e., decayed) by TTP (i.e., ZFP36) as indicated in our transcriptome analysis (Table 1, Figure 3). In fact, TTP plays a major role in the decay of cytokines and immediate early response gene transcripts (Amit et al., 2007; Tiedje et al., 2016), and we have previously reported its induction in response to GS isolate SPs (Ma'ayeh et al., 2017). These findings suggest an interesting scenario wherein some inflammatory cytokines are produced by parasitized IECs initiating an inflammatory response but this response is simultaneously modulated by the parasite/parasite SPs possibly to avoid excessive inflammation.

Changes in gene transcription varied between differentiated and proliferating Caco-2 cells. Results from qPCR indicated similar or slightly higher inflammatory responses in proliferating relative to differentiated IECs. This is in line with a previous study showing a higher general inflammatory response in proliferating Caco-2 cells in an experiment mimicking inflammatory responses using a combination of stimuli (TNF-α, IL1-β, INF-γ, and LPS) (Van De Walle et al., 2010). The authors reasoned that differentiated IECs acquire a hypo-responsiveness state (lower expression of toll like receptors) due to constant exposure to products of the microbial flora (Van De Walle et al., 2010). Differentiated Caco-2 cells are similar to small intestinal enterocytes located at the top of intestinal villi whereas proliferating Caco-2 are similar to non-differentiate cells in the crypts (Park et al., 2015). *Giardia* can colonize both the villi and the crypts (Owen et al., 1979) and our results suggest that there are differences in the responses to *Giardia* depending on where the parasites attach.

The production of ROS and nitric oxide (NO) by host cells (Figure 3) represents the first lines of defense against intestinal pathogens (Roxström-Lindquist et al., 2006). Differentiated Caco-2 cells produce ROS in response to *G. intestinalis* (WB and GS) (Ma'ayeh et al., 2015) and they up-regulate *nox1* and *inos* upon exposure to WB isolate (Roxström-Lindquist et al., 2005). The induction of these genes was also seen in this study, moreover, we identified DTGs whose functions are required to protect cells from oxidative damage. This suggests that while IECs mount an oxidative attack against trophozoites, they also prepare a defense mechanism against free radicals. *Giardia* anti-oxidative defenses, on the other hand, are capable of neutralizing host cells ROS (Ma'ayeh et al., 2015), leading to the persistence of trophozoites on cell surface during infection.

*Giardia* infections in known to induce apoptosis both in *in vivo* and *in vitro* (Chin et al., 2002; Panaro et al., 2007; Troeger et al., 2007). Using WB isolate and HCT-8 intestinal epithelial cell line, it has been shown that the induction of apoptosis is mediated through both the intrinsic and extrinsic apoptotic pathways (Panaro et al., 2007). Another report also showed that the induction of apoptosis is strain-dependent (Chin et al., 2002). Here, DTGs enrichment analysis indicated the induction of both apoptotic pathways (Figure 3). Although we demonstrated PARP cleavage to confirm the induction of apoptosis in IECs by GS trophozoites, further investigations are warranted in this context, as this is likely to be a combination of multiple factors including extrinsic activation (e.g., via parasite cysteine proteases) (Piña-Vázquez et al., 2012), glucose starvation (Yu et al., 2008), arginine depletion (Roxström-Lindquist et al., 2005), oxidative stress (Roxström-Lindquist et al., 2005) and the disruption of intestinal epithelial barrier integrity (Buret, 2007; Troeger et al., 2007).

*Giardia* is known to arrest the cell cycle in IECs (Stadelmann et al., 2012, 2013). In *in vitro* interactions with WB isolate, this effect has been linked to arginine depletion, reduced polyamine levels and up-regulated cell cycle inhibitory genes (Stadelmann et al., 2012). To our knowledge, this is the first report of such an effect in interactions with GS isolate (Figure 3). Although we did not test for arginine depletion or the reduction of polyamine levels, we showed that the occurrence of DNA damage (1.5–4.5 h) could be another factor contributing to cell cycle arrest. In line with a previous result showing cell cycle arrest at the G1/S upon exposure to WB trophozoites (Stadelmann et al., 2012), we further showed that cell cycle arrest occurred at the M phase in co-incubations with GS, indicating a general effect on cell cycle. Based on the transcriptomic results, other factors like nutrients depletion (e.g., glucose), oxidative stress (Figure 3) or the induction of apoptosis may also contribute to cell cycle arrest.

The results from our transcriptome analysis showed that GS isolate colonization could affect the integrity of tight junctions, the microvilli and the mucus layer at the enterocyte surface (Figure 3). It is known that GS infection delocalizes the tight junctions claudin-1 and occludin in polarized Caco-2 cells (Humen et al., 2011) but herein, we further show effects on *amot* (scaffolding protein) and *mpp5* (PDZ protein that binds tight junction proteins, also known as Pals1) (Table 1). AMOT binds a protein called rich1, upon which they are targeted to a protein complex at the tight junctions containing MPP5 (among other proteins) (Roh et al., 2003; Wells et al., 2006). This association maintains the tight junctions' complex stability, and the down-regulation of *amot* and *mpp5* herein (if this manifest at the protein level) might indicate the opposite. *Giardia* also attaches strongly to enterocytes affecting the structure of microvilli. CDHR5 is an intermicrovillar adhesion molecule that forms, via its extracellular domain, calcium-dependent heterophilic complexes with CDHR2 on adjacent microvilli (Crawley et al., 2014). These complexes control the packing of microvilli at the apical membrane of epithelial cells and thus, the up-regulation of *cdhr2* upon exposure to GS might indicate a response to counteract the damage inflicted upon microvilli. Lastly, a recent study showed that infection with GS isolate depletes mucus throughout the small and large intestines, in human biopsies and in mice, and indices *muc2* and *muc5* gene expression in mice (Amat et al., 2017). The depletion of mucus was found to be a combinatory effect of mucus hypersecretion and direct breakdown by parasite cysteine proteases. Despite the difference in infection model, the up-regulation of *muc2* gene shown here was consistent with that in mice model and demonstrates a general effect of GS isolate infection on the mucus layer.

In a previous report, we showed that *Giardia* and host cells secrete the same types of metabolic proteins during interactions, indicating competition for acquiring nutrients (Ma'ayeh et al., 2017). Here, parasitized IECs transcriptome displayed transcriptional up-regulation of amino acids transporters and lipid metabolic enzymes (Figure 3), corroborating the above finding. *Giardia* relies on exogenous sources for acquiring amino acids (Samra et al., 1987), reducing the amounts available for IECs. In fact, arginine depletion by the parasite represents the best example on this effect (Stadelmann et al., 2012). Experiments *in vivo* further showed a reduced uptake of L-phenyl-alanine, L-lysine and L-aspartic acid in the intestines of infected swiss albino mice and a significant drop in the transport of L-alanine and glycine in mice (Anand et al., 1982). *Giardia* also scavenges the gut lumen for lipids including cholesterol (Pham et al., 2017), and secretes enzymes involved in phospholipids remodeling and scavenging fatty acids (Ma'ayeh et al., 2017). Cholesterol, specifically, is required for lipid raft formation and cholesterol starvation induces encystation (Yichoy et al., 2011; Mendez et al., 2015). Therefore, it is plausible to think that IECs up-regulate lipid metabolic enzymes and transporters to outcompete their consumption by GS trophozoites.

In summary, GS isolate infection in differentiated Caco-2 cells up-regulated a wide range of genes involved in inflammatory signaling, cell cycle regulation, attenuation of oxidative stress, induction of apoptosis, and maintaining the microvilli structure and the mucin layer covering the enterocytes (Figure 3). Exposure to trophozoites selectively induced the phosphorylation of ERK1/2, and the nuclear recruitment of NFκB and AP-1 whereas P38 phosphorylation was reduced. Assemblage B (e.g., GS isolate) SPs were capable of degrading inflammatory cytokines, suggesting an attenuation of inflammatory responses during infection. The degradation of cytokines mRNA, via RNA binding proteins like TTP is another suggested mechanism for the regulation of inflammation during infection. The parasitized IECs transcriptome suggests a modulated uptake of glucose, amino acids and sodium ions during infection together with cell cycle arrest and the induction of apoptosis, with direct relevance to the pathology of giardiasis. Compared to a previous study using the same model of infection (i.e., differentiated Caco-2 cells)(Roxström-Lindquist et al., 2005), this study has provided novel and more insights into the disease mechanisms induced by an assemblage B isolate. Further studies will focus on using more complex systems such as animal models and enteroids to explore host responses to *Giardia* infection in its natural habitat (i.e., small intestines).

## Authors contributions

SM and SS conceptualization. SM and SS experimental design. SM, SS, LK, and KS performing experiments and analyzing results. AG, BA, and AJ bioinformatic support. SM and SS writing the Manuscript. BA and AJ proofreading the manuscript.

### Conflict of interest statement

The authors declare that the research was conducted in the absence of any commercial or financial relationships that could be construed as a potential conflict of interest.

## Supplementary material

Figure S1 Differentially transcribed genes (DTGs) in the colon adenocarcinoma cell line, Caco-2, upon interaction with *Giardia intestinalis* GS isolate for 1.5h, 3, and 4.5 h. **(A)** Venn diagram showing the number of overlapping or specific DTGs to the three interaction time points **(B)** Correlation of Log_2_ fold change in RNA levels between RNA sequencing and quantitative real time PCR (qPCR) **(C)** Log_2_ fold change in RNA levels of genes encoding inflammatory cytokines or associated with immune signaling, response to cytokines or mRNA decay of cytokines.

Figure S2 Heat map representing the fold change of RNA levels in the colon adenocarcinoma cell line, Caco-2, in both differentiated (D) and proliferative (P) states upon interaction with *Giardia intestinalis* GS isolate for 1.5, 3, and 4.5 h. The heat map displays a general higher fold change in the group of selected genes for differentiated Caco-2 cells.

Figure S3 Immunofluorescence images showing the nuclear recruitment of nuclear factor kappa-B (NFκB) in the differentiated colon adenocarcinoma cell line, Caco2, upon co-incubation with *Giardia intestinalis* GS isolate trophozoites. The green fluorescent dots in the nucleus represent NFκB.

Figure S4 The translocation of nuclear factor kappa-B (NFκB) into the nucleus of proliferating colon carcinoma cell line, Caco-2, upon co-incubation with *Giardia intestinalis* GS isolate. Immune staining shows green fluorescent dots in the nucleus representing NFκB. Proliferating Caco-2 cells incubated with 100 ng of tumor necrosis factor alpha per ml of culture medium represent the positive control in this experiment. Negative control is proliferating Caco-2 cells incubated alone in culture medium.

Figure S5 Heat map showing temporal changes in the RNA levels of genes associated with response to oxidative stress and cell cycle regulation in the differentiated colon carcinoma cell line, Caco-2, co-incubated with *Giardia intestinalis* GS isolate. The fold change in RNA levels is presented for three co-incubation time points (1.5, 3, and 4.5 h).

Table S1 Differentially transcribed genes (DTGs) in the differentiated colon adenocarcinoma cell line, Caco-2, in response to interaction with *Giardia intestinalis* isolate, GS, for 1.5 h. Note that significantly up-regulated genes are in red font whereas significantly down-regulated genes are in blue font (adjusted *P* < 0.05).

Table S2 Differentially transcribed genes (DTGs) in the differentiated colon adenocarcinoma cell line, Caco-2, in response to interaction with *Giardia intestinalis* isolate, GS, for 3 h. Note that significantly up-regulated GENES are in red font whereas significantly down-regulated genes are in blue font (adjusted *P* < 0.05).

Table S3 Differentially transcribed genes (DTGs) in the differentiated colon adenocarcinoma cell line, Caco-2, in response to interaction with *Giardia intestinalis* isolate, GS, for 4.5 h. Note that significantly up-regulated genes are in red font whereas significantly down-regulated genes are in blue font (adjusted *P* < 0.05).

Table S4 Differentially transcribed genes (DTGs) in the differentiated colon adenocarcinoma cell line, Caco-2, in response to interaction with *Giardia intestinalis* isolate, GS. DTGs were identified upon a comparison of Caco-2 cells transcriptomes at 3h versus 1.5h. Note that significantly up-regulated genes are in red font whereas significantly down-regulated genes are in blue font (adjusted *P* < 0.05).

Table S5 Differentially transcribed genes (DTGs) in the differentiated colon adenocarcinoma cell line, Caco-2, in response to interaction with *Giardia intestinalis* isolate, GS. DTGs were identified upon a comparison of Caco-2 cells transcriptomes at 4.5 vs. 3 h. Note that significantly up-regulated genes are in red font whereas significantly down-regulated genes are in blue font (adjusted *P* < 0.05).

Table S6 Differentially transcribed genes (DTGs) in the differentiated colon adenocarcinoma cell line, Caco-2, incubated with *Giardia intestinalis* GS isolate for 1.5, 3, and 4.5 h. The table shows overlapping genes in the three time points (orange-colored cell) and DTGs specific to each interaction time points (1.5 h green, 3 blue and 4.5 h yellow).

Table S7 Gene ontology (GO) analysis for the differentially transcribed genes (DTGs) in the differentiated colon adenocarcinoma cell line, Caco-2, upon interaction with *Giardia intestinalis* GS isolate for 1.5 h. The table shows an enrichment of GO functions associated with biological process (BP), molecular process (MP) and cellular component (CC) (*P* < 0.05).

Table S8 Gene ontology (GO) analysis for the differentially transcribed genes (DTGs) in the differentiated colon adenocarcinoma cell line, Caco-2, upon interaction with *Giardia intestinalis* GS isolate for 3 h. The table shows an enrichment of GO functions associated with biological process (BP), molecular process (MP) and cellular component (CC) (*P* < 0.05).

Table S9 Gene ontology (GO) analysis for the differentially transcribed genes (DTGs) in the differentiated colon adenocarcinoma cell line, Caco-2, upon interaction with *Giardia intestinalis* GS isolate for 4.5 h. The table shows an enrichment of GO functions associated with biological process (BP), molecular process (MP) and cellular component (CC) (*P* < 0.05).

Table S10 Gene ontology (GO) analysis for the differentially transcribed genes (DTGs) in the differentiated colon adenocarcinoma cell line, Caco-2, upon interaction with *Giardia intestinalis* GS isolate. The GO terms are enriched upon comparing Caco2 cell transcriptome between 3 and 1.5 h. The table shows an enrichment of GO functions associated with biological process (BP), molecular process (MP) and cellular component (CC) (*P* < 0.05).
