# Corrigendum: Responses of the Differentiated Intestinal Epithelial Cell Line Caco-2 to Infection With the *Giardia intestinalis* GS Isolate

**DOI:** 10.3389/fcimb.2018.00297

**Published:** 2018-08-24

**Authors:** Showgy Y. Ma'ayeh, Livia Knörr, Karin Sköld, Alexandra Garnham, Brendan R. E. Ansell, Aaron R. Jex, Staffan G. Svärd

**Affiliations:** ^1^Department of Cell and Molecular Biology, Uppsala University, Uppsala, Sweden; ^2^Population Health & Immunity Division, The Walter and Eliza Hall Institute of Medical Research, Parkville, VIC, Australia; ^3^Faculty of Veterinary Science, The University of Melbourne, Parkville, VIC, Australia

**Keywords:** host-parasite interaction, *Giardia*, transcriptome, cell cycle, apoptosis

An author name was incorrectly spelled as **Alexandra Granham**. The correct spelling is **Alexandra Garnham**. The authors apologize for this error and state that this does not change the scientific conclusions of the article in any way.

The original article has been updated.

## Conflict of interest statement

The authors declare that the research was conducted in the absence of any commercial or financial relationships that could be construed as a potential conflict of interest.

