# Supplementary figures and images for: Responses of the Differentiated Intestinal Epithelial Cell Line Caco-2 to Infection With the Giardia intestinalis GS Isolate
Source: Front Cell Infect Microbiol. 2018 Jul 16;8:244. doi: 10.3389/fcimb.2018.00244 (PMC6055019; doi:10.3389/fcimb.2018.00244)

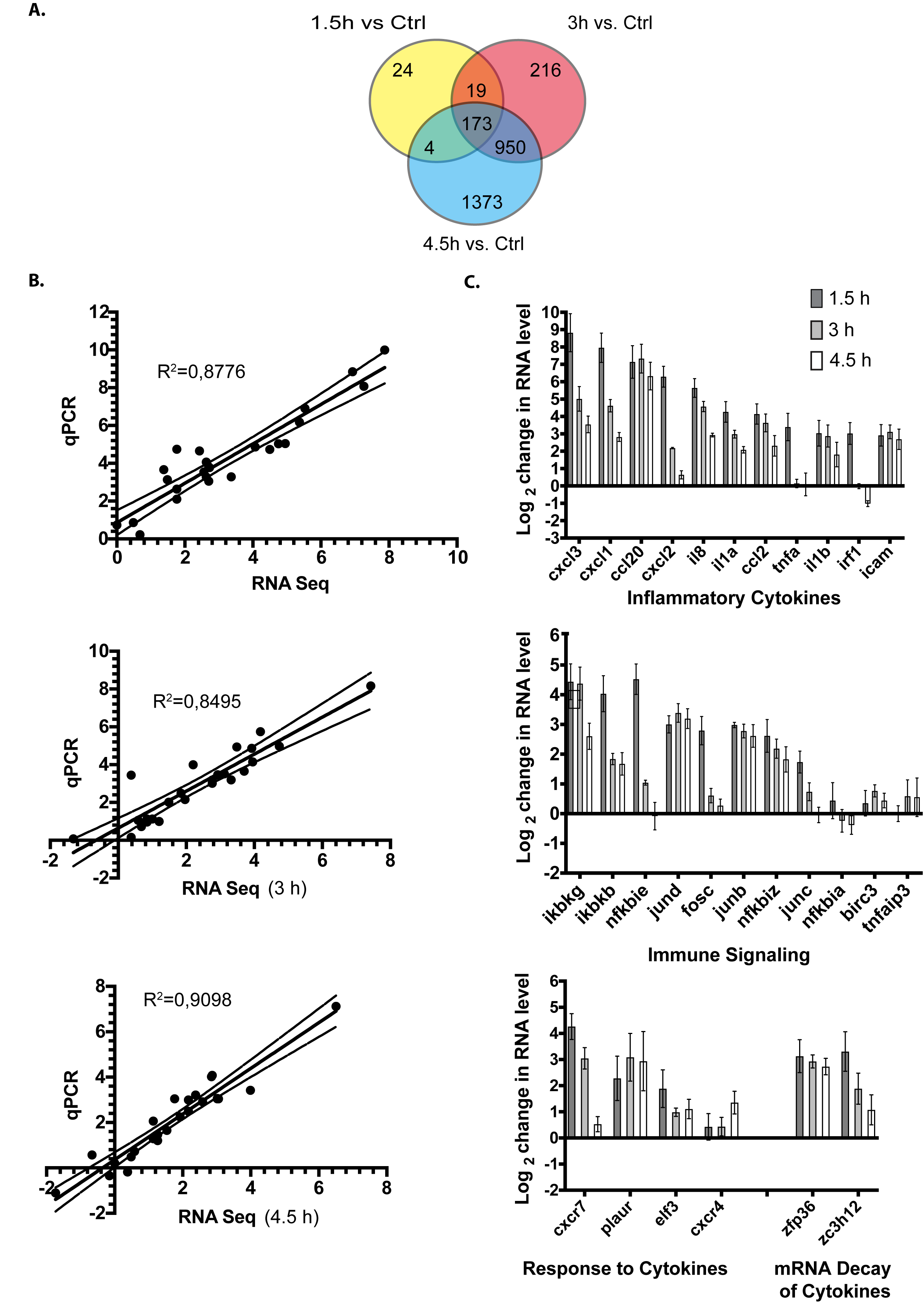

Supplement: Figure S1 — Differentially transcribed genes (DTGs) in the colon adenocarcinoma cell line, Caco-2, upon interaction with Giardia intestinalis GS isolate for 1.5h, 3, and 4.5 h. (A) Venn diagram showing the number of overlapping or specific DTGs to the three interaction time points (B) Correlation of Log2 fold change in RNA levels between RNA sequencing and quantitative real time PCR (qPCR) (C) Log2 fold change in RNA levels of genes encoding inflammatory cytokines or associated with immune signaling, response to cytokines or mRNA decay of cytokines. [file Image_1.TIF]

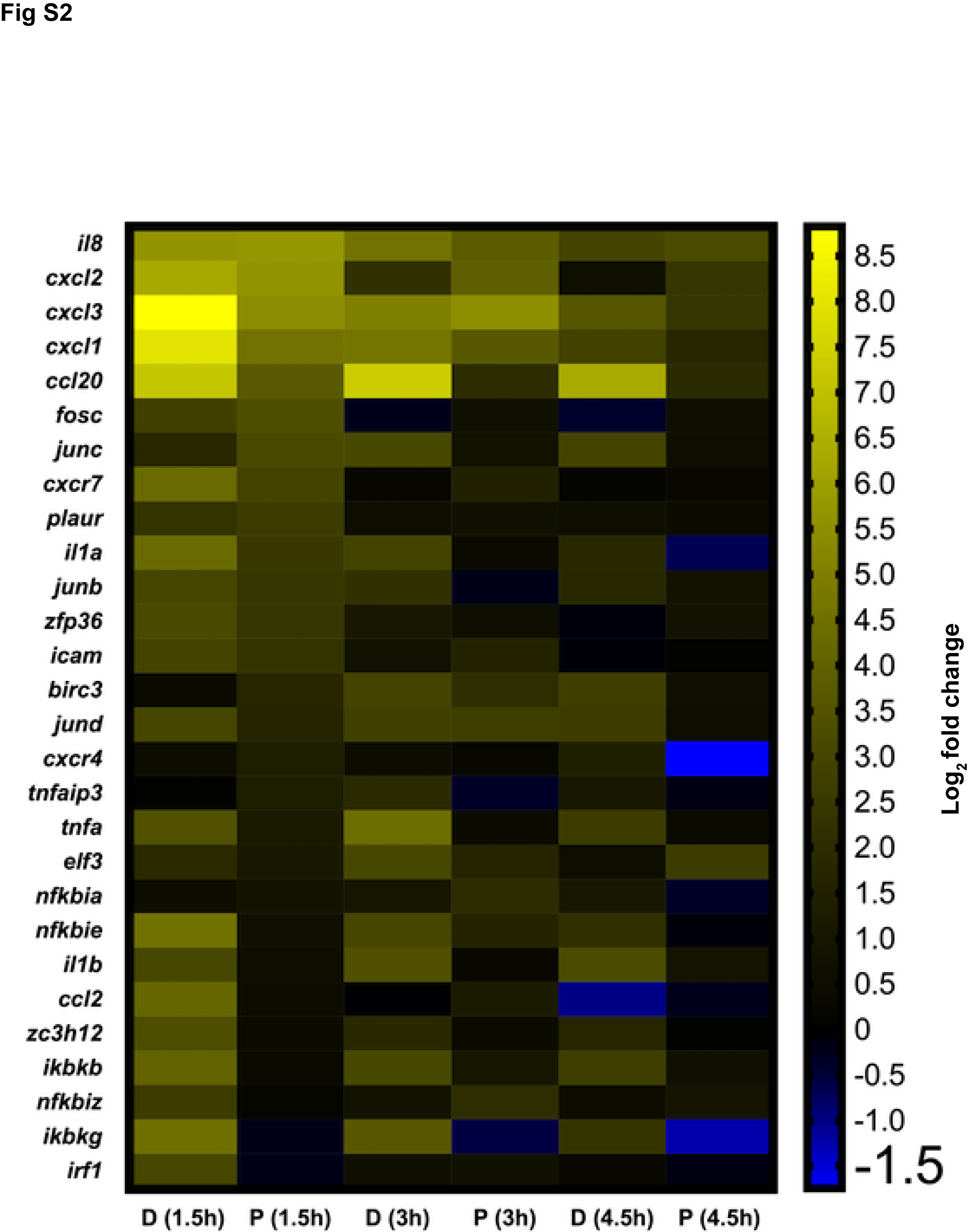

Supplement: Figure S2 — Heat map representing the fold change of RNA levels in the colon adenocarcinoma cell line, Caco-2, in both differentiated (D) and proliferative (P) states upon interaction with Giardia intestinalis GS isolate for 1.5, 3, and 4.5 h. The heat map displays a general higher fold change in the group of selected genes for differentiated Caco-2 cells. [file Image_2.TIF]

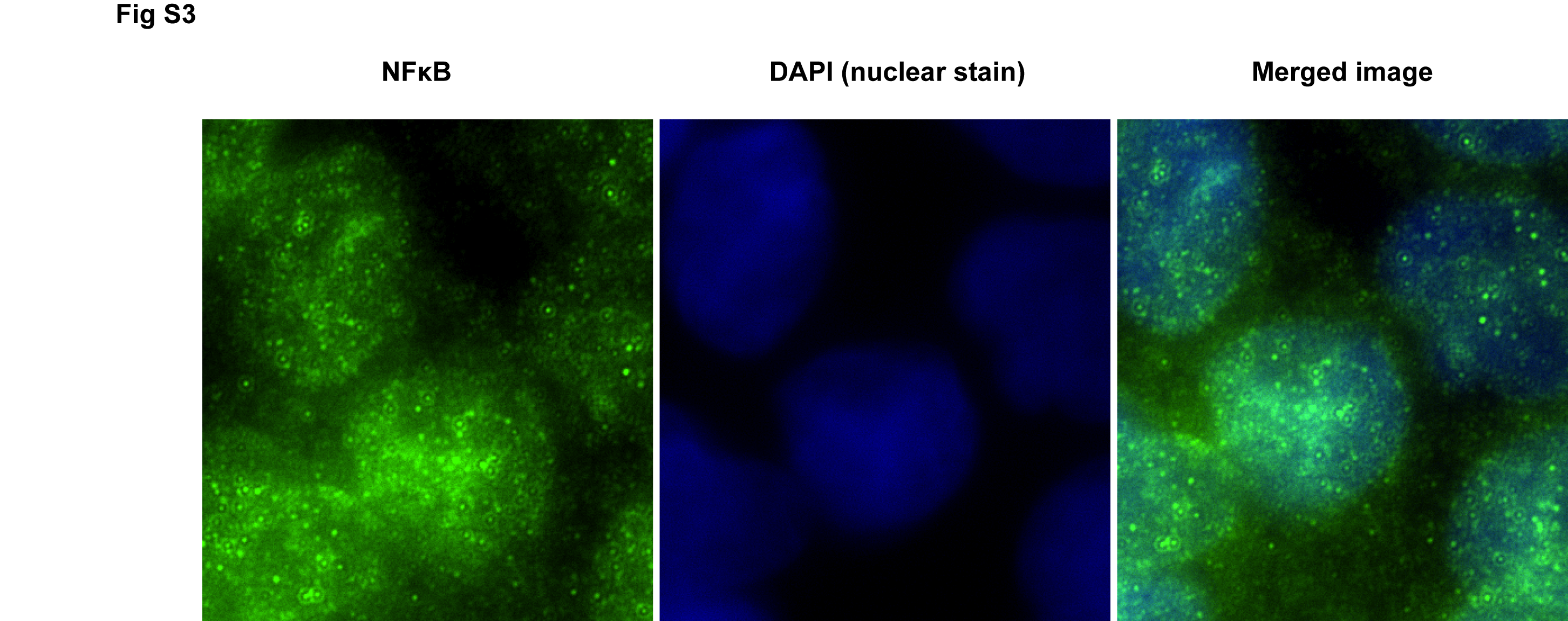

Supplement: Figure S3 — Immunofluorescence images showing the nuclear recruitment of nuclear factor kappa-B (NFκB) in the differentiated colon adenocarcinoma cell line, Caco2, upon co-incubation with Giardia intestinalis GS isolate trophozoites. The green fluorescent dots in the nucleus represent NFκB. [file Image_3.TIF]

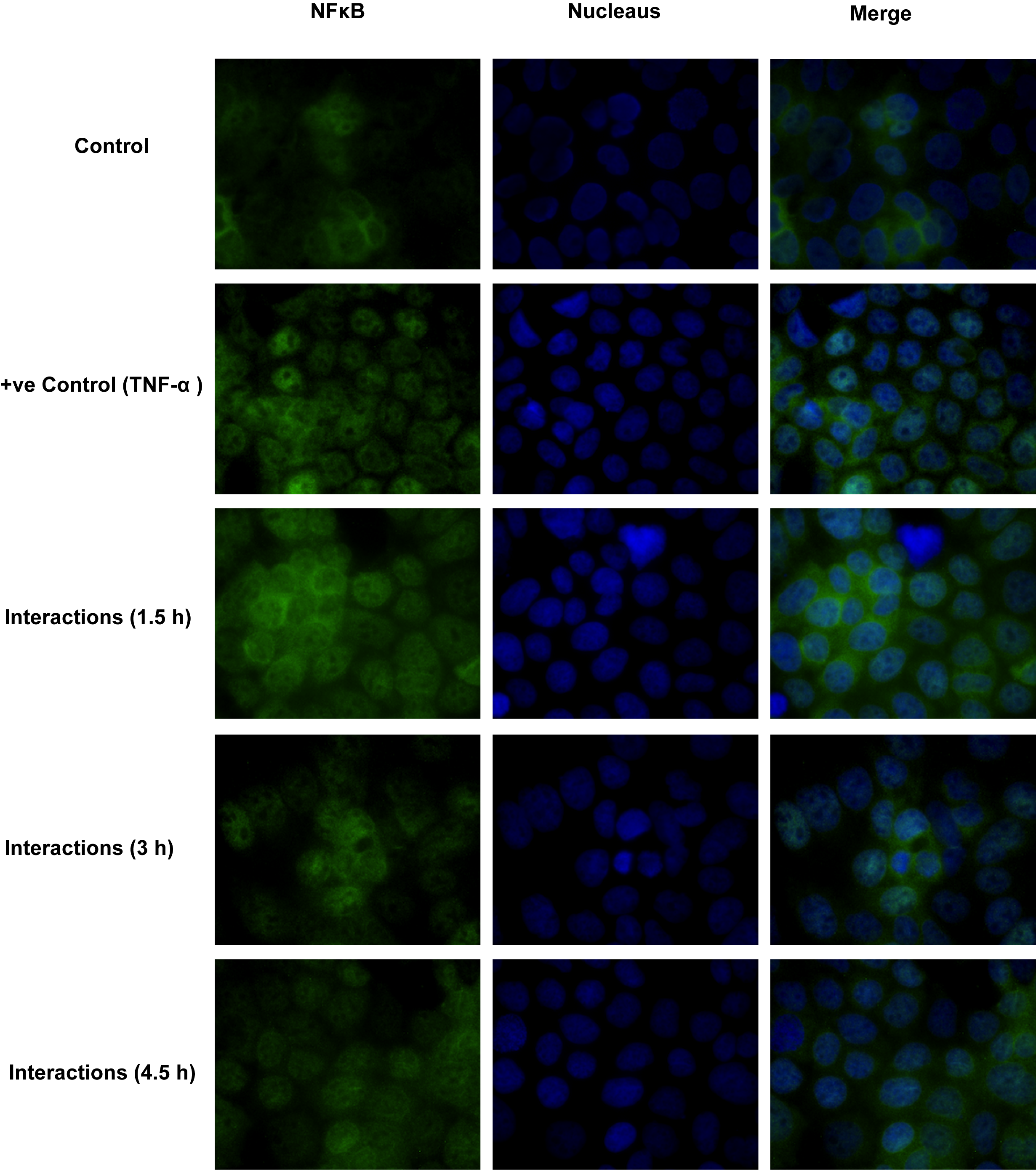

Supplement: Figure S4 — The translocation of nuclear factor kappa-B (NFκB) into the nucleus of proliferating colon carcinoma cell line, Caco-2, upon co-incubation with Giardia intestinalis GS isolate. Immune staining shows green fluorescent dots in the nucleus representing NFκB. Proliferating Caco-2 cells incubated with 100 ng of tumor necrosis factor alpha per ml of culture medium represent the positive control in this experiment. Negative control is proliferating Caco-2 cells incubated alone in culture medium. [file Image_4.TIF]

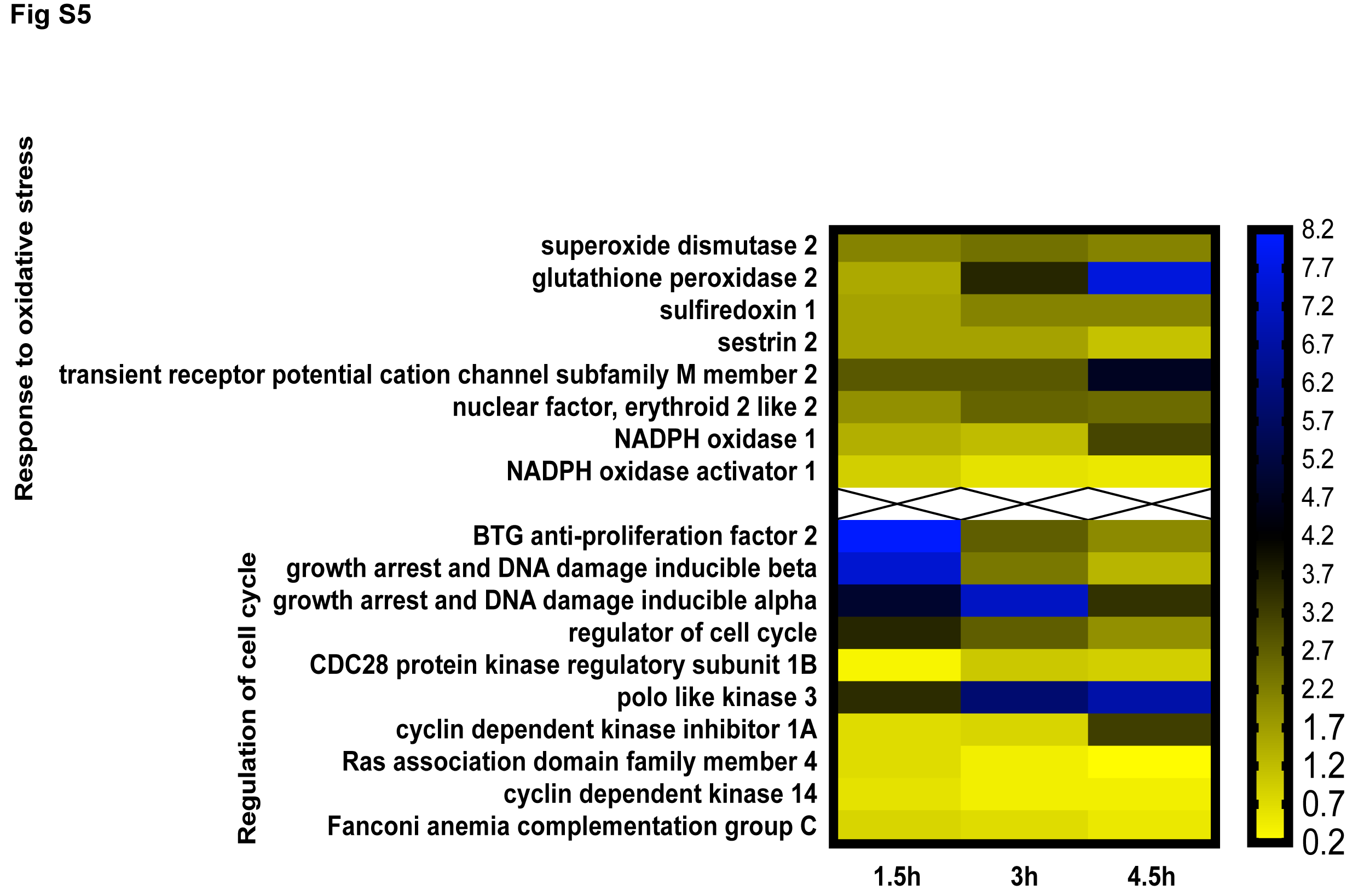

Supplement: Figure S5 — Heat map showing temporal changes in the RNA levels of genes associated with response to oxidative stress and cell cycle regulation in the differentiated colon carcinoma cell line, Caco-2, co-incubated with Giardia intestinalis GS isolate. The fold change in RNA levels is presented for three co-incubation time points (1.5, 3, and 4.5 h). [file Image_5.TIF]
